# Gene networks and transcriptional regulators associated with liver cancer development and progression

**DOI:** 10.1186/s12920-021-00883-5

**Published:** 2021-02-04

**Authors:** Tatiana Meier, Max Timm, Matteo Montani, Ludwig Wilkens

**Affiliations:** 1 Institute of Pathology, Nordstadtkrankenhaus, Hanover, Germany; 2 Clinic for Laryngology, Rhinology and Otology, Medical School Hanover, Hanover, Germany; 3 Institute of Pathology, University of Bern, Bern, Switzerland; 4 Institute of Human Genetics, Medical School Hanover, Hanover, Germany

**Keywords:** HCC, Chromosomal instability, Dedifferentiation, Gene expression, Transcription factors, Genomics, Transcriptomics

## Abstract

**Background:**

Treatment options for hepatocellular carcinoma (HCC) are limited, and overall survival is poor. Despite the high frequency of this malignoma, its basic disease mechanisms are poorly understood. Therefore, the aim of this study was to use different methodological approaches and combine the results to improve our knowledge on the development and progression of HCC.

**Methods:**

Twenty-three HCC samples were characterized by histological, morphometric and cytogenetic analyses, as well as comparative genomic hybridization (aCGH) and genome-wide gene expression followed by a bioinformatic search for potential transcriptional regulators and master regulatory molecules of gene networks.

**Results:**

Histological evaluation revealed low, intermediate and high-grade HCCs, and gene expression analysis split them into two main sets: GE1-HCC and GE2-HCC, with a low and high proliferation gene expression signature, respectively. Array-based comparative genomic hybridization demonstrated a high level of chromosomal instability, with recurrent chromosomal gains of 1q, 6p, 7q, 8q, 11q, 17q, 19p/q and 20q in both HCC groups and losses of 1p, 4q, 6q, 13q and 18q characteristic for GE2-HCC. Gene expression and bioinformatics analyses revealed that different genes and gene regulatory networks underlie the distinct biological features observed in GE1-HCC and GE2-HCC. Besides previously reported dysregulated genes, the current study identified new candidate genes with a putative role in liver cancer, e.g. *C1orf35*, *PAFAH1B3*, *ZNF219* and others.

**Conclusion:**

Analysis of our findings, in accordance with the available published data, argues in favour of the notion that the activated E2F1 signalling pathway, which can be responsible for both inappropriate cell proliferation and initial chromosomal instability, plays a pivotal role in HCC development and progression. A dedifferentiation switch that manifests in exaggerated gene expression changes might be due to turning on transcriptional co-regulators with broad impact on gene expression, e.g. POU2F1 (OCT1) and NFY, as a response to accumulating cell stress during malignant development. Our findings point towards the necessity of different approaches for the treatment of HCC forms with low and high proliferation signatures and provide new candidates for developing appropriate HCC therapies.

## Background

Hepatocellular carcinoma (HCC) is the sixth most prevalent cancer and the second most lethal tumour worldwide; its incidence continues to rise [1]. Prognosis and treatment options for HCC are poor and mainly dependent on tumour stage [2] and histological grade, which reflects tumour biology [3]. High-grade HCC can develop from low-grade HCC in a course of several months in 75% of patients [4]. Notably, cancer tissues with different histological grades can be frequently found in individual HCC nodules [5], a phenomenon that provides a ‘nodule-in-nodule appearance’. It may be assumed by these histological findings that there is a stepwise development from low- to high-grade HCC based on molecular mechanisms that are still unknown. Likewise, a stepwise increase in chromosomal instability and aneuploidy occurs during progression from well differentiated to moderately or low differentiated HCC [6]. Chromosomal instability predicts drug resistance and poor prognosis in multiple cancer types, but the causes of chromosomal instability in HCC and other tumour types are still poorly understood [7, 8]. By gene expression profiling, HCC may be divided into two main groups: one with upregulation of genes responsible for cell proliferation and anti-apoptosis and poor outcome, and the other with the opposite findings [9, 10].

Although a number of studies have been performed using individual analytical methods, a combined approach for the investigation of HCC at histological, genomic and gene expression levels with a bioinformatics search for transcriptional regulators and master regulators of signal transduction pathways is rare. Therefore, the aim of our study was to gain a closer insight into the network of histological alterations, chromosomal aberrations and gene expression profiles and apply bioinformatics tools to find possible key players in the process of malignant transformation. For this purpose, we analysed 23 HCC samples of different histological grades and adjacent non-tumourous (NT) liver tissues with regard to the above-mentioned aspects. With this approach, we identified different genes and gene regulatory networks linked to distinct biological features observed in HCCs with low and high proliferative profiles. These findings provide additional candidate genes for the development of novel, subtype-dependent options for HCC therapy. Based on our results and comprehensive analysis of available published data, we also hypothesise key molecular events that underlie HCC onset and progression.

## Methods

### Tumour samples

The samples for this study comprised tissue from partial hepatectomy of 15 HCC patients treated at the Department of Abdominal Surgery of the Inselspital (Bern, Switzerland) in 2002–2008 (clinicopathological data in Additional file 1). Twenty-three tumour samples were collected from these surgical specimens, as well as 17 NT samples adjacent to the carcinoma nodules. Tissue samples were formalin fixed and paraffin embedded following standard procedures. Edmonson–Steiner grading [11] was applied using haematoxylin and eosin (H&E) stained slides. For molecular analyses, tumours and paired NT tissues were macro-dissected after defining the regions of interest by an experienced pathologist to ensure a content of tumour cells of at least 75%.

### Gene expression analyses and data evaluation

Total RNA was isolated using the Qiagen AllPrep DNA/RNA FFPE kit strictly according to the manufacturer’s protocol. DNA was checked for contamination with qPCR using Brilliant SYBRGreen QPCR Master Mix (Stratagene) and qPCR normalisation primers for single-copy regions of non-coding genomic DNA from the SideStep II Cell Lysis Analysis Kit (Agilent). From the kit, Primer Set 1 (233 bp) and Primer Set 2 (244 bp) were used, and the PCR cycling program was 95 °C for 10 min followed by 40 cycles of 95 °C for 30 s, 60 °C for 30 s, and 72 °C for 30 s. PCR reactions were run on a Versant kPCR real-time PCR instrument (Siemens) with MxPro™ QPCR Software for the Mx3000P/3005P QPCR System (Stratagene). Analysis of RNA quality was performed using an Agilent RNA 6000 Nano Assay on an Agilent 2100 Bioanalyzer. RNA integrity numbers for the isolated RNAs were 2.1–2.5.

Further procedures strictly followed the Gene Expression FFPE Workflow v. 2.0.1 protocol developed by Agilent Technologies. Briefly, a complementary DNA (cDNA) library was generated from the total RNA using the TransPlex™ Whole Transcriptome Amplification Kit (Sigma). cDNA was labelled with Cy3 using the Genomic DNA ULS Labeling Kit (Agilent) and purified with the Agilent KREApure column. Next, 500 ng of Cy3-cDNA was hybridized to one-colour Agilent SurePrint G3 Gene Expression v2 8 × 60 K Microarray (G4851B). The array contained 50,559 probes covering over 40,000 transcripts, including lncRNAs and transcripts of unknown coding potential (TUCPs). Hybridization, washing, and scanning procedures were performed exactly as described in the One-Colour Microarray-Based Gene Expression Analysis Protocol, v. 6.6 (Agilent). Washed arrays were immediately scanned on the Agilent C scanner and Agilent Feature Extraction software v. 11.0.1.1 was used to extract signal intensities from images.

Microarray data evaluation was performed using the United States Food and Drug Administration’s genomic tool ArrayTrack with embedded PCA and HCA [12]. Raw gene expression values were normalized using the 75^th^ percentile of the overall signal value, as recommended by the manufacturer, and log_2_ transformed prior to further statistical and bioinformatic analyses. Genes with significant differences in their expressions were identified by one-way ANOVA in multiple-group testing, or by unpaired Welch’s *t* test in pair-wise comparisons. For multiple probe sets for one gene, the median value was calculated.

For functional analysis of differently expressed genes, we employed Pathway Enrichment Analysis implemented in ArrayTrack which is based on canonical pathways supplied by the KEGG database. Fisher’s exact test was applied to identify biological pathways with statistically significant enrichment in regulated genes, the cut off being *P* ≤ 0.05.

### Bioinformatics search for transcriptional regulators and master regulators of gene networks

To identify potential transcriptional regulators and master regulator molecules, we applied promoter analysis and upstream network analysis of differentially expressed genes provided by the GeneXplain platform (https://genexplain.com/) with integrated MATCH™ tool [13], TRANSFAC® [14] and TRANSPATH® [15] databases (release 2.4.1).

Gene promoters were defined as sequences from − 1000 to + 100 relative to the transcription start sites (Ensemble database). Promoter sequences of co-regulated genes were searched for potential transcription factor binding sites (TFBSs) using MATCH™ tool and TRANSFAC® library of positional weight matrices. The frequency of putative TFBSs in the genes differentially expressed in tumours (Yes set) was compared to the corresponding value for the default set of 300 human housekeeping genes of which expression is kept unchanged (No set). Transcription factors with overrepresented binding sites in the Yes set versus No set (ratio > 1) were considered as potential regulators.

Master regulator molecules in the signal transduction pathways upstream of the dysregulated genes were identified using the curated database of pathways and protein interactions TRANSPATH® with default parameters. Molecules with the lowest Rank Sum values were considered as the most likely candidates.

### Real-time polymerase chain reaction (qPCR)

For validation of gene expression data obtained with microarrays, we employed qPCR for selected genes. Equal amounts of cDNAs from 12 NTs, 8 GE1-HCCs and 12 GE2-HCCs were pooled into the corresponding groups, and 10–20 ng of each cDNA pool were used to perform three independent qPCR experiments, each experiment in triplicate. We used a Versant kPCR real-time PCR instrument (Siemens) with the MxPro™ QPCR Software for Mx3000P/3005P QPCR System (Stratagene). PCR assays were performed for *MKI67, HJURP, KPNA2, XPOT* and *TSNARE1* (internal control) with predesigned TaqMan® Gene Expression Assays and the TaqMan® Gene Expression Master Mix (Applied Biosystems) according to the manufacturer’s protocol. Assays for non-coding RNAs *H19, HNF-AS1, SNAR-A3, SNAR-B2, SNAR-H* were performed with Brilliant II SYBR® Green QPCR Low Rox Master Mix (Agilent) according to the manufacturer’s ‘Recommended Protocol with Two-Step Cycling (All Targets)’ with subsequent ‘Dissociation Program for All Targets’. The *SNAR* gene primers were derived from Parrott et al. [16]. We designed *H19*, *HNF-AS1 and TSNARE1* (an internal control) primers with the NCBI Primer-BLAST tool (https://www.ncbi.nlm.nih.gov/tools/primer-blast/) and purchased them from Biomers (Ulm, Germany). The sequences are as follows: *SNAR-A3*, F 5′-AGCCATTGTGGCTCAGGC-3′, R 5′-TTTTCCGACCCATGTGGACC-3′; *SNAR-B2*, F 5′-GCCATTGTGGCTCCGGC-3′, R 5′-AACCCATGTGGACCAGGTTG-3′; *SNAR-H*, F 5′-CCACTGTGGCTCCGGC-3′, R 5′-AATGTGGACCAGGTTGGCCT-3′; *H19*, F 5′-GACGTGACAAGCAGGACATGA-3′, R 5′-TAAGGTGTTCAGGAAGGCCG-3′; *HNF1A-AS1*, F 5′-ACTAAAATTCGGGCGAGGCA-3′, R 5′-GACTGGCTGAAGGGACACTC-3′; and *TSNARE1*, F 5′-GAAGAAAATTGCAGAAAAGTCCAGA-3′, R 5′-GTCACTCCCGTTAAAGACCTTC-3′. All PCR products were checked with an agarose gel. We used the comparative threshold cycle method (ΔΔCt) to quantify the relative amount of product transcripts. ∆Ct values = ((Ct target gene)—(Ct reference gene)) from three independent experiments were evaluated with *t* test to identify the statistically significant differences between the means of the sample groups. We calculated the FC for each gene as 2^−∆∆Ct values^, where ΔΔCt = ΔCt_HCC _− ΔCt_NT_.

### Array-based comparative genomic hybridisation (aCGH)

aCGH was performed exactly as previously described [17]; the detailed protocol is also freely accessible in the ArrayExpress database under E-MTAB-8886. Cy5-labelled genomic DNA from tissue samples and Cy3-labeled human male genomic DNA (Promega, Germany) as a reference were hybridised to Agilent G4450A arrays. We normalised, visualised and analysed data with the Agilent Genomic Workbench v7.0 software using ADM-2 algorithm. We set cut-offs for chromosomal amplifications/deletions to the minimum number of probes in aberration interval ≥ 3 and average absolute log_2_ ratio ≥ 0.2, which corresponds to the presence of single copy aberration in about one third of analysed cell population.

### Nuclear size measurement

Nuclear size measurement was done using Leica Application Suite, v.3.7.0 on 2-µM thick H&E-stained tissue sections in the same tumour and non-tumour regions used for molecular analyses. At least 100 cells were measured.

Fluorescence in situ hybridisation (FISH).

FISH was performed on 4 µm thick tissue sections using locus-specific probes for centromeres, CEP1, CEP3, CEP7, CEP8, and CEP17 (all supplied by Abbott, Wiesbaden, Germany) according to the supplier’s instructions. An Axio Imager microscope (Zeiss, Jena, Germany) was used for evaluation of fluorescent signals. A total of 100 nuclei were evaluated in each sample for calculation of the mean value.

## Results

### Gene expression analysis divided HCC into two distinct groups: GE1 and GE2

**Fig. 1:**
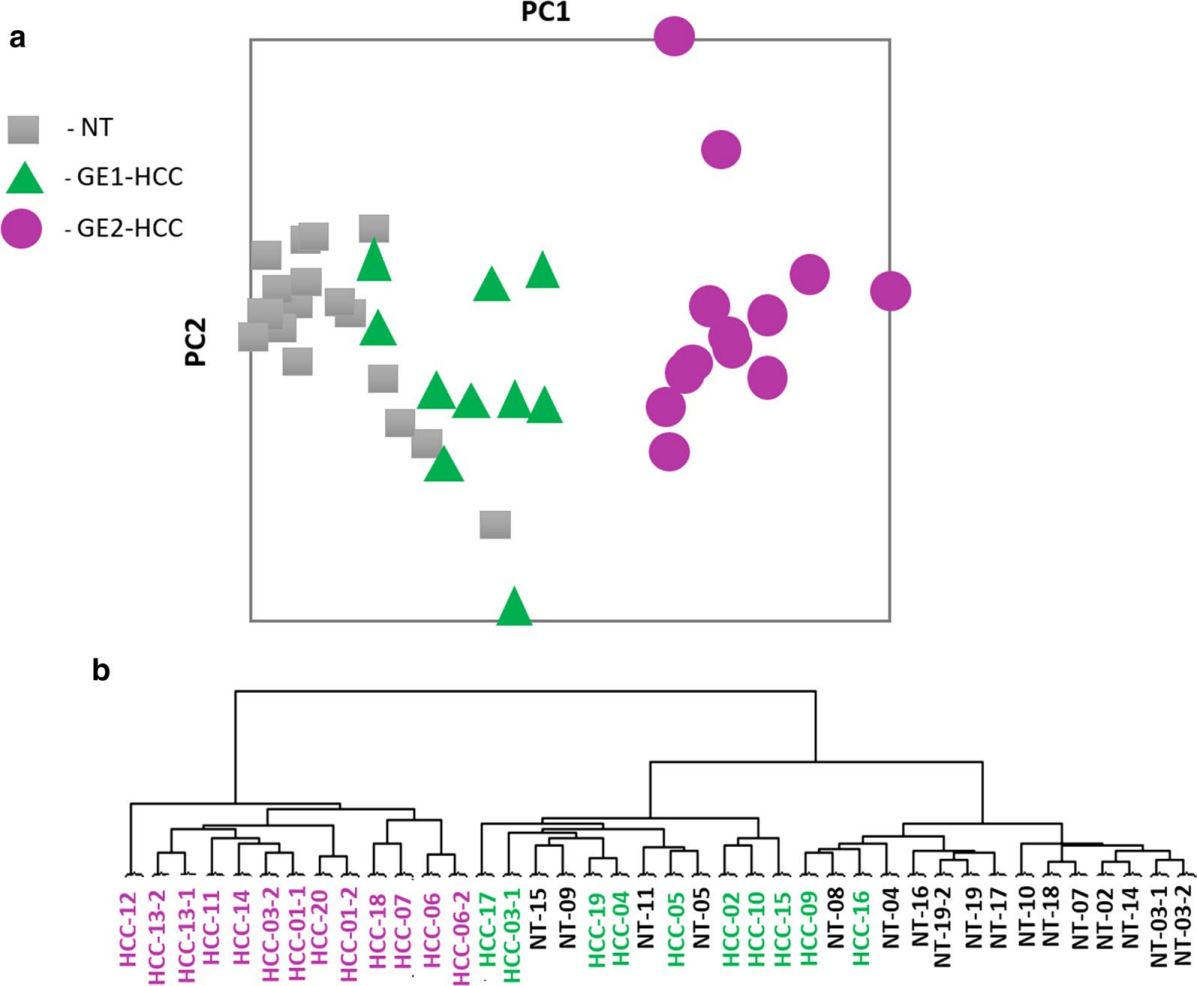
Two distinctive subtypes of hepatocellular carcinoma (HCC) were defined by **a** principal component analysis and **b** hierarchical cluster analysis. Gene expression values were analysed from 23 HCC samples of various histological grade, as well as 17 surrounding non-tumourous (NT) liver samples. We selected probe sets differentially expressed between 23 HCC samples and 17 NT samples, using a mean absolute ratio ≥ 2 and false discovery rate ≤ 0.05 for the analyses. Based on the results of the analyses, 10 HCC samples with gene expression patterns similar to NT tissues were assigned to the GE1-HCC subgroup, while 13 HCC samples that clearly demonstrated a different gene expression pattern from both GE1-HCC and NT samples were assigned to the GE2-HCC subgroup

Analysis of 23 HCC and 17 NT samples (Additional file 1) yielded 458 differently expressed probes with a mean absolute fold change (FC) ≥ 2 and false discovery rate (FDR) ≤ 0.05. These probes represented 321 unique genes: 131 were upregulated and 190 were downregulated in tumours (Additional file 2). Principal component analysis (PCA) (Fig. 1a) and hierarchical clustering analysis (HCA) (Fig. 1b) based on the gene expression of 458 probes divided all samples into two main groups of 13 HCC samples and another group of 10 HCC and 17 NT samples. The full image for HCA analysis with gene expression values is shown in Additional file 3.

Gene expression patterns of 10 HCC samples (GE1-HCC) were rather similar to the NT tissues. In contrast, gene expression pattern of 13 HCCs (GE2-HCC) clearly differed from GE1-HCC and adjacent NT tissues. After analyses, one tumour sample, HCC-17, was re-classified as a cholangiocellular carcinoma (CCC). The expression profile of this sample was very close to GE1-HCC.

To identify genes specifically deregulated in the two HCC subtypes, we performed three pair-wise *t* tests—GE1-HCC versus NT, GE2-HCC versus NT and GE2-HCC vs GE1-HCC—and multiple comparison of GE1-HCC, GE2-HCC and NT groups using one-way analysis of variance (ANOVA). The cut-offs for a significant change in gene expression for GE2-HCC versus NT and GE2-HCC versus GE1-HCC were FDR ≤ 0.05 and mean absolute FC ≥ 2. For the GE1-HCC versus NT comparison, we applied a less stringent cut-off, namely *P* ≤ 0.01, instead of a more stringent FDR threshold to recover enough differentially expressed genes for further analyses. All genes identified in the three pairwise *t* tests showed a significant change in a multiple-group testing (*P* ≤ 0.05), with the exception of two genes. Additional files 4–6 list the details for each gene.

**Fig. 2:**
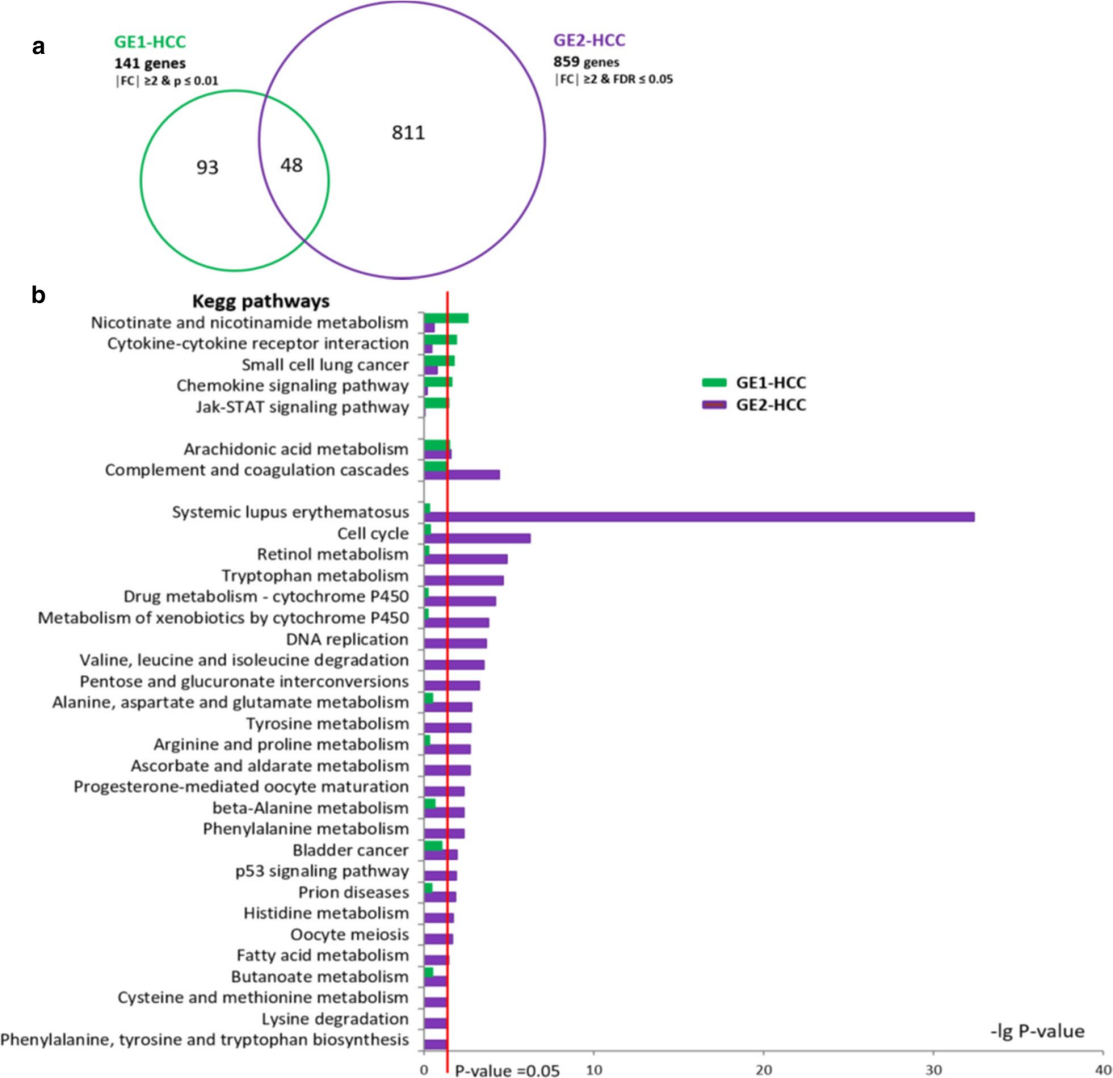
Comparison of differentially expressed genes and deregulated pathways in the GE1 and GE2 hepatocellular carcinoma (HCC) subgroups. **a** The two gene lists resulting from the pair-wise *t* tests—10 GE1-HCC versus 17 NT and 13 GE2-HCC versus 17 NT—were compared via Venn Diagram analysis. **b** Pathway enrichment analysis was performed using the same two gene lists, and the Kyoto Encyclopedia of Genes and Genomes (KEGG) was used to identify pathways with the significant enrichment in the deregulated genes (Fisher’s *P* value ≤ 0.05) in GE1 and GE2 HCC subgroups

In general, there was a strong perturbation in gene expression with regard to the number of regulated genes as well as expression fold changes in the GE2-HCC group. In contrast, in GE1-HCC there were only moderate gene expression changes and far fewer differentially regulated genes compared with surrounding NT tissue. In GE1-HCC, there were 209 differentially expressed probes with a mean absolute FC in the range 2 to 4.9, despite applying the less stringent *P* value cut-off. These probes represented 141 unique genes: 26 upregulated and 115 downregulated. In GE2-HCC, 1254 probes were differentially regulated in comparison with NT adjacent tissues, with an absolute FC from 2 to 31.7. They represented 859 unique genes: 395 upregulated and 464 downregulated. Analysis of the differentially expressed genes in tumours showed that a part of them were significantly regulated in both groups of tumours (Fig. 2a).

The direction of the common gene expression changes, i.e. activation or repression, was concordant in both tumour groups, with the exception of long non-coding RNA H19, which was repressed in GE1-HCC and increased in GE2-HCC. Differentially expressed genes in GE1-HCC and GE2-HCC were further used for analyses of functional pathways, gene networks and gene promoters. Pathway enrichment analysis (Fisher’s exact test) revealed five pathways specifically deregulated in GE1-HCC (Fig. 2b). In contrast, 26 pathways were affected in GE2-HCC. The dysregulated pathways in GE1-HCC were linked to immune response and NAD metabolism, both reported to be involved in cancer [18, 19]. The prominent feature of GE2-HCC was strong perturbation in the cell cycle, in particular DNA replication and the p53 signalling pathway. Additionally, cytochrome P450–catalysed drug metabolism, retinoic acid metabolism, fatty acid metabolism, cysteine and methionine metabolism and the metabolism of several other amino acids were specifically affected in GE2-HCC. Pathways related to cancer and immune diseases were also significantly changed in GE2-HCC. There were only two commonly regulated pathways in both HCC subgroups: complement and coagulation cascades and arachidonic acid metabolism. These pathways are involved in immune defence and inflammation and reportedly have a significant role in the development and progression of numerous malignancies [20, 21]. In general, the functional analysis of genes regulated in tumours and associated pathways indicated that GE2-HCC tumours exhibited a high proliferative capacity and strongly perturbed cell metabolism. They likely represent a much more aggressive phenotype compared with GE1-HCC tumours.

### Array-based comparative genomic hybridisation (aCGH) revealed deletions to be typical for GE2-HCC

Numerous chromosomal aberrations were identified, including both gains and losses, which were shared by 30–81.8% of the HCC samples; some of these chromosomal aberrations were also found in the adjacent non-tumorous tissue (Table 1, Additional files 7–8). There were no frequently occurring deletions in NT samples, but some gains, e.g., 7q, 8q, 11p, and 19p, were observed in 50%-69.2% of non-tumorous tissues, derived from both GE1 and GE2 groups.

**Table 1:** Chromosomal aberrations in hepatocellular carcinoma (HCC) tumours compared to adjacent non-tumourous tissues (NT)

Chr	Arm	Type of aberration		Significant^a^	Non-neoplastic tissues (NT)		HCC	
		NT	HCC		Size^b^ of intervals	% cases affected	Size^b^ of intervals	% cases affected
**1**	**p**	**–**	**Loss**	**Yes**	**0**	**0**	**407**	**27.3–31.8**
**1**	**q**	**–**	**Gain**	**Yes**	**2108**	**7.7–23.1**	**2108**	**54.5–77.3**
2	q	**–**	Gain	Yes	0	0	206	27.3**–**36.4
3	p	**–**	Gain	Yes	173	15.4–23.1	173	54.5**–**73.6
**4**	**q**	**–**	**Loss**	**Yes**	**0**	**0**	**689**	**27.3–31.8**
5	p	**–**	Gain	Yes	0	0	12	27.3
5	q	**–**	Loss	Yes	0	0	673	27.3**–**31.8
5	q	**–**	Gain	Yes	0	0	154	27.3
**6**	**p**	**–**	**Gain**	**Yes**	**525**	**7.7–15.4**	**1061**	**31.8–68.2**
**6**	**q**	**–**	**Loss**	**Yes**	**0**	**0**	**26**	**27.3**
7	p	**–**	Gain	Yes	100	7.7–15.4	810	27.3–68.2
*7*	*q*	*Gain*	*Gain*	*Yes*	*96*	*23.1–30.8*	*146*	*36.4–72.7*
*7*	*q*	*Gain*	*Gain*	*No*	*94*	*30.7–53.8*	*94*	*63.6–72.7*
**8**	**p**	**–**	**Loss**	**Yes**	**103**	**7.7**	**525**	**31.8–63.6**
**8**	**q**	**–**	**Gain**	**Yes**	**14**	**7.7**	**1456**	**27.3–50**
*8*	*q*	*Gain*	*Gain*	*Yes*	*22*	*46.2*	*22*	*81.8*
*8*	*q*	*Gain*	*Gain*	*No*	*54*	*38.4–69.2*	*54*	*72.7–81.8*
9	q	**–**	Gain	Yes	10	15.4	10	63.6
*9*	*q*	*Gain*	*Gain*	*No*	*276*	*30.7*	*276*	*63.6*
10	q	**–**	Gain	Yes	12	7.7	51	27.3–50
10	q	**–**	Loss	Yes	0	0	13	27.3
*11*	*q*	*Gain*	*Gain*	*Yes*	*80*	*38.5*	*80*	*77.3*
*11*	*p/q*	*Gain*	*Gain*	*No*	*373*	*38.4–53.8*	*373*	*59.1–72.7*
12	p	**–**	Gain	Yes	0	0	38	36.4
12	q	**–**	Gain	Yes	323	15.4	323	54.5–63.6
**13**	**q**	**–**	**Loss**	**Yes**	**23**	**7.7**	**996**	**27.3–50.0**
13	q	**–**	Gain	Yes	21	7.7	21	45.5–50.0
14	q	**–**	Gain	Yes	158	7.7–23.1	158	45.5–59.1
15	q	**–**	Gain	Yes	38	15.4	498	27.3–54.5
16	p	**–**	Gain	Yes	221	7.7	221	40.9–50.5
**16**	**q**	**–**	**Loss**	**Yes**	**15**	**7.7**	**15**	**31.8**
**17**	**p/q**	**–**	**Gain**	**Yes**	**1802**	**7.7–15.4**	**1802**	**40.9–63.6**
18	q	**–**	Loss	Yes	0	0	759	27.3–36.4
19	q	**–**	Gain	Yes	10	7.7	10	45.5
*19*	*p*	*Gain*	*Gain*	*No*	*652*	*53.8*	*652*	*68.2–77.3*
*19*	*q*	*Gain*	*Gain*	*No*	*999*	*30.8–46.2*	*999*	*63.6–68.2*
**20**	**p/q**	**–**	**Gain**	**Yes**	**820**	**7.7–23.1**	**1125**	**31.8–63.6**
21	q	**–**	Gain	Yes	0	0	235	27.3–36.4
22	q	**–**	Gain	Yes	810	7.7–15.4	810	59.1–63.6

The table only includes aberrations that spanned more than 10 probes. Known chromosomal imbalances in HCC are marked in bold. Common aberrations found in both HCC (*n* = 22) and adjacent NT (*n* = 17) samples were included when they affected > 50% of cases in one tissue set and > 30% of cases in the other; they are presented in italics

*Chr* chromosome

^a^Differential aberrations between HCC and NT samples were considered statistically significant when *P* < 0.05 in differential aberration analysis

^b^The interval size is expressed as the total number of probes that spanned the aberration interval

When analysing the GE1-HCC and GE2-HCC subgroups separately, obvious differences in their genomic patterns are observed (Table 2, Additional files 9–10). Most gains were found repeatedly in both the GE1-HCC and GE2-HCC subgroups. In contrast, losses were typical for the GE2-HCC subgroup, in which they occurred in 40–80% of the samples. Some loci, e.g., 1p, 16q, and 17p, which demonstrated recurrent gains in GE1 samples showed recurrent losses in the GE2-HCC subgroup. In the GE1-HCC subgroup, the only repeatedly identified loss was at 8p, which was shared by 30–50% of the samples.

**Table 2:** Chromosomal aberrations in hepatocellular carcinoma (HCC) subgroups with a low (GE1) and high (GE2) proliferation gene expression profile

Chr	Arm	Type of aberration		Significant^a^	HCC GE1		HCC GE2	
		GE1	GE2		Size^b^ of intervals	% cases affected	Size^b^ of intervals	% cases affected
1	p	Gain	–	Yes	175	50	116	8.3
1	p	Gain	Loss	No	885	30–50	885	33.3–41.7
*1*	*q*	*Gain*	*Gain*	*No*	*2214*	*50–80*	*2214*	*41.7–83.3*
*2*	*p/q*	*Gain*	*Gain*	*No*	*92*	*50*	*92*	*33.3*
*3*	*p/q*	*Gain*	*Gain*	*No*	*195*	*50–70*	*252*	*33.3–66.7*
4	q	–	Loss	Yes	0	0	783	41.7– 50.0
5	p	–	Loss	Yes	0	0	106	41.7
5	q	Gain	–	Yes	207	50	207	8.8
5	q	Gain	Loss	No	215	30–40	215	33.3
*6*	*p*	*Gain*	*Gain*	*No*	*474*	*50–60*	*474*	*58.3–75*
6	q	Gain	Loss	No	34	30	34	33.3
7	p/q	Gain	–	Yes	2111	40–70	915	8.3–25.0
*7*	*q*	*Gain*	*Gain*	*Yes*	*34*	*90*	*34*	*41.7*
*7*	*p/q*	*Gain*	*Gain*	*No*	*298*	*60–90*	*298*	*33.3–58.3*
*8*	*p*	*Loss*	*Loss*	*No*	*286*	*30–50*	*286*	*58.3–75*
8	p	Gain	Loss	No	55	30–50	28	41.7–50
*8*	*q*	*Gain*	*Gain*	*No*	*1364*	*50–100*	*1364*	*33.3–66.7*
*9*	*p*	*Gain*	*Gain*	*No*	*34*	*50*	*34*	*33.3*
*9*	*q*	*Gain*	*Gain*	*No*	*292*	*80*	*292*	*50*
*10*	*q*	*Gain*	*Gain*	*No*	*16*	*60*	*16*	*41.7*
*11*	*p/q*	*Gain*	*Gain*	*No*	*169*	*60–80*	*169*	*41.7–50*
*11*	*q*	*Gain*	*Gain*	*Yes*	*400*	*100*	*400*	*50–58.3*
12	p	Gain	–	Yes	85	60	85	16.7
*12*	*q*	*Gain*	*Gain*	*Yes*	*188*	*80*	*188*	*33.3*
*12*	*p/q*	*Gain*	*Gain*	*No*	*213*	*50–80*	*213*	*33.3–50*
13	q	–	Loss	Yes	568	10	1199	41.4–83.3
13	q	Gain	–	Yes	11	70	11	25
*13*	*q*	*Gain*	*Gain*	*No*	*10*	*70*	*10*	*33,3*
*14*	*q*	*Gain*	*Gain*	*No*	*191*	*40–70*	*191*	*33.3–58.3*
*15*	*q*	*Gain*	*Gain*	*No*	*43*	*50*	*43*	*58.3*
16	p/q	Gain	–	Yes	721	50–60	706	8.3–16.7
*16*	*p*	*Gain*	*Gain*	*No*	*119*	*60*	*119*	*33.3–41.7*
*16*	*q*	*Gain*	*Loss*	*Yes/no*	*157*	*50*	*157*	*33.3–50*
17	p	Gain	Loss	Yes*/no*	541	60–80	541	33.3–50
*17*	*q*	*Gain*	*Gain*	*No*	*1452*	*40–50*	*1452*	*41.5–75*
18	q	–	Loss	Yes	149	10	149	58.3
18	q	–	Loss	No^c^	668	10	668	50
*19*	*p*	*Gain*	*Gain*	*Yes*	*762*	*100*	*762*	*41.7–58.3*
*19*	*q*	*Gain*	*Gain*	*Yes*	*39*	*70–90*	*39*	*16.7–41.7*
*19*	*q*	*Gain*	*Gain*	*No*	*1030*	*80–90*	*1030*	*50*
*20*	*p/q*	*Gain*	*Gain*	*No*	*841*	*30–60*	*841*	*50–66.7*
21	q	–	Loss	Yes	0	0	91	41,7
21	q	Gain	–	Yes	43	70	43	25
*22*	*q*	*Gain*	*Gain*	*No*	*829*	*70–80*	*43*	*50*

The table only includes aberrations that spanned more than 10 probes. Similar aberrations found in both HCC subgroups were included when they affected > 50% cases in one tissue set and > 30% cases in the other; they are presented in italics. Aberrations that presented changes in the opposite direction in the HCC subgroups were included when the aberration incidence in each subgroup was ≥ 30%

*Chr* chromosome

^a^We considered differential aberrations between the two HCC groups to be statistically significant when *P* < 0.05 in differential aberration analysis

^b^The interval size is expressed as the total number of probes that spanned the aberration interval

^c^The *P* value for differential loss of 18q in HCC-GE2 was 0.06

Some amplified chromosomal loci encode genes with tumour-promoting functions that were strongly upregulated in tumours, as denoted by gene expression analysis (Table 3).

**Table 3:** Selected upregulated genes with oncogenic functions located in amplified chromosomal regions

Gene	Chr locus	Amplified in GE1-HCC, %	Amplified in GE2-HCC, %	Amplified in NT, %	Mean fold change^a^ of gene expression in GE1-HCC versus NT	Mean fold change^a^ of gene expression in GE2-HCC versus NT
*C1orf35*	1q42.13	80	75	15.4	2.15	2.4
*HIST2 cluster*	1q21	80	75	15.4	No change	2.8–22.2
*PARP1*	1q42.12	70	66.7	15.4	No change	2.06
*KPNA2*	17q24.2	50	75	7.7	No change	2.63
*BIRC5*	17q25.3	50	75	7.7	3.41	34.5
*TK1*	17q25.3	50	75	7.7	1.69	6.93
*E2F1*	20q11.2	60	66.7	15.4	1.97	6.43
*KIFC1*	6p21.3	60	66.7	15.4	No change	9.44
*HIST1 cluster*	6p21-6p22	60	66.7	15.4	No change	2.6–15.9
*UBE2C*	20q13.12	60	66.7	15.4	1.64	20.35
*RECQL4*	8q24	100	66.7	69.2	2.12	6.7
*H19*	11p15.5	80	41.7	53.8	− 3.06	4.58
*SNAR-A3*	19q13.33	90	50	46.2	No change	7.72
*SNAR-B2*	19q13.33	90	50	46.2	No change	15.83
*SNAR-D*	19q13.33	90	50	46.2	No change	17.5
*SNAR-G2*	19q13.33	90	50	46.2	No change	17.45
*ILF3*	19q13.2	90	50	46.2	No change	2.21
*PAFAH1B3*	19q13.1	90	50	46.2	No change	3.85

*Chr* chromosome, *HCC* hepatocellular carcinoma, *NT* non-tumourous

^a^Only statistically significant fold changes are shown

### Nuclear size was significantly increased in tumours, most notably in GE2-HCC

**Fig. 3:**
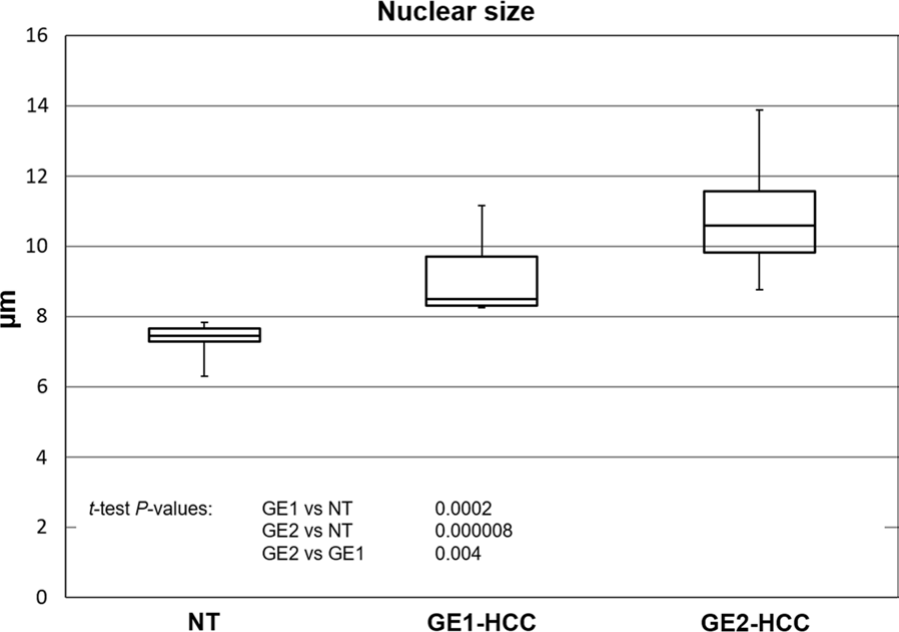
Nuclear sizes in the GE1 and GE2 hepatocellular carcinoma (HCC) subtypes and non-tumourous (NT) tissue. Nuclear size (µM) was determined in NT tissue (n = 10), as well as in two distinctive subgroups of HCC that were defined by gene expression profiling, GE1 (n = 10) and GE2 (n = 11); 100 cells per sample were evaluated. The box plot diagram shows the distribution of nuclear sizes in each tissue group, where the horizontal line demonstrates median nuclear size, the boxes span the interquartile range, and the “antennas” represent the minimum and maximum observed values

Nuclear size, which is an important diagnostic criterion for tumour grading, was significantly larger in all HCC samples when compared to the adjacent NT tissues (Fig. 3). The median nuclear size in NT tissues was 7.44 µM, which falls in the range reported for the average normal hepatocyte nucleus of 7–8 µM [22]. Meanwhile, the median nuclear size values in the GE1-HCC and GE2-HCC subgroups fell above this range, 8.49 µM and 10.50 µM, respectively. The interquartile ranges were 0.4 µM, 1.4 µM, and 1.8 µM for the NT, GE1-HCC and GE2-HCC groups, respectively, indicating a much more variable nuclear size in the HCC groups.

### GE2-HCC showed high-level aneuploidy

Fluorescence in situ hybridisation (FISH) for the five selected chromosomes revealed a mean number of signals in GE1-HCC close to NT samples, with the highest value of 2.73 (Table 4). In contrast, GE2-HCC, which typically also had a higher histological grade and enlarged nuclei compared with GE1-HCC, were highly aneuploid, showing a variable increased number of chromosomes (up to 5.44).

**Table 4:** Fluorescence in situ hybridisation (FISH) analyses of selected chromosomes and histological grade in GE1-HCC and GE2-HCC

Samples	Mean number of FISH signals per cell*					Gene expression HCC subgroup	Histological grade (Edmonson)
	CEP1	CEP3	CEP7	CEP8	CEP17		
HCC-03-1	1.96	1.60	1.44	1.54	2.05	GE1	1
HCC-09	1.96	2.73	2.29	2	2	GE1	1–2
HCC-02	1.74	1.81	1.75	2.19	1.89	GE1	2
HCC-04	1.85	1.71	1.77	1.87	1.81	GE1	2
HCC-15	1.87	2.36	2.32	1.9	1.89	GE1	2
HCC-16	2.01	2.29	1.99	2.13	1.99	GE1	2
HCC-17	1.76	1.59	1.69	1.68	1.75	GE1	2
HCC-19	1.63	1.86	1.89	1.72	1.64	GE1	2
HCC-05	1.66	1.80	1.61	2.05	1.77	GE1	2–3
HCC-10	2.57	2.33	2.48	1.87	1.92	GE1	2–3
Mean GE1	1.90	2.01	1.92	1.90	1.87		
SD GE1	0.27	0.39	0.34	0.21	0.13		
HCC-03-2	1.86	1.57	1.74	1.76	2.01	GE2	2
HCC-01-1	1.82	1.59	1.69	1.79	1.83	GE2	2–3
HCC-07	4.12	2.87	2.73	2.48	3.4	GE2	2–3
HCC-12	3.88	4.59	3.45	4.44	4.49	GE2	2–3
HCC-13	3.48	2.12	2.63	3.72	4.3	GE2	2–3
HCC-18	–	3.43	3.39	2.76	–	GE2	2–3
HCC-20	1.97	3.27	2.34	3.21	2.14	GE2	2–3
HCC-06–2	2.13	2.8	2.58	2.6	2.03	GE2	3
HCC-11	2.09	2.61	2.54	2.48	1.93	GE2	3–4
HCC-14	4.2	4.59	4.55	5.44	4.21	GE2	3–4
Mean GE2	2.84	2.94	2.76	3.07	2.93		
SD GE2	1.05	1.07	0.85	1.17	1.15		

*HCC* hepatocellular carcinoma, *SD* standard deviation

*100 cells were counted

Notably, histological grading and molecular group assignment did not match completely. Whereas all grade 1 HCC samples were assigned to GE1-HCC, based on gene expression profiling, and all grade 3 or 4 HCC samples were allotted to GE2-HCC, grade 2 tumours were in both molecular groups. Furthermore, GE2-HCC included more samples with a hepatitis background, although there was no pivotal difference between the two groups. Overall, 77% of GE2-HCC and 56% of GE1-HCC samples (1 sample had unknown status) were derived from patients with positive hepatitis serology.

### Validation of microarray gene expression changes by real-time polymerase chain reaction (qPCR)

Microarray gene expression data were confirmed by qPCR analyses for selected genes and non-coding RNAs that were overexpressed in the GE2-HCC subgroup. (Table 5). There was good concordance between qPCR and microarray data for most genes; in particular, the strong up regulation of these genes in GE2 HCC was confirmed. In a number of cases, the fold changes in gene expression obtained via the qPCR method were much larger than those identified via microarray analyses, possibly due to the higher sensitivity of the qPCR assay, especially in cases of genes expressed at low levels. For example, in the case of both *MKI67* and *HJURP*, microarray analysis revealed significant increases in expression in the GE2-HCC subgroup only, whereas qPCR analysis detected upregulation in both of the HCC subgroups, although a much higher relative level was detected in the GE2-HCC subgroup as well.

**Table 5:** Validation of microarray gene expression data by real-time polymerase chain reaction (qPCR) analysis

Comparison	Gene	Microarrays		qPCR	
		Mean FC	*P*	Mean FC	*P*
GE1-HCC versus NT	*MKI67*	1.77	0.092	4.92	2.30E-04
	*HJURP*	1.27	0.32	15.84	2.60E-05
	*KPNA2*	1.28	0.253	0.96	0.756
	*XPOT*	1.02	0.949	0.78	0.041
	*HNF1A-AS1*	3.25	0.007	3.19	0.019
	*H19*	0.33	0.006	0.13	0.006
	*SNAR-A3*	1.08	0.813	0.61	0.03
	*SNAR-B2*	0.58	0.305	0.26	0.01
	*SNAR-H*	0.68	0.427	0.92	0.752
GE2-HCC versus NT	*MKI67*	12.96	0	36.64	5.30E-05
	*HJURP*	7.17	0	25.91	1.10E-05
	*KPNA2*	2.63	0	12.81	0.004
	*XPOT*	2.54	0.001	2.26	0.001
	*HNF1A-AS1*	5.27	0	7.05	0.026
	*H19*	4.58	0.002	3.1	0.002
	*SNAR-A3*	7.72	0	24.2	0.002
	*SNAR-B2*	15.83	0	47.5	0.001
	*SNAR-H*	14.05	0	29.55	0.001
GE2-HCC versus GE1-HCC	*MKI67*	7.32	0	7.52	8.40E-05
	*HJURP*	5.63	0	1.68	0.013
	*KPNA2*	2.05	0.001	13.42	0.001
	*XPOT*	2.5	0.003	2.92	1.20E-04
	*HNF1A-AS1*	1.65	0.204	2.21	0.026
	*H19*	14.01	0	23.81	0.002
	*SNAR-A3*	8.33	0	39.58	0.002
	*SNAR-B2*	27.26	0	181.02	0.001
	*SNAR-H*	20.66	0	32.22	0

*FC* fold change, *HCC* hepatocellular carcinoma, *NT* non-tumourous

### Master regulator molecules of gene networks in GE1-HCC and GE2-HCC

Master regulator molecules in the signal transduction pathways, at a distance of up to 10 steps upstream of the dysregulated genes, were identified for up- and downregulated genes in the GE1-HCC and GE2-HCC subgroups (Table 6, Additional files 11–14) using the curated database of pathways and protein interactions, TRANSPATH®, integrated in the GeneXplain platform. The top three master regulators identified for the 26 genes upregulated in the GE1-HCC subgroup included two cell-surface proteins involved in pro-survival signaling in the liver and E2F1, a key regulator of the cell cycle. Moderate and strong overexpression of E2F1 was observed in the GE1-HCC and GE2-HCC subgroups, respectively. The top three master regulator molecules identified for the 395 upregulated genes in the GE2-HCC subgroup comprised essential regulators of progression through the mitotic phase of the cell cycle, and overexpression of the genes coding these proteins was specifically observed in the GE2-HCC subgroup.

**Table 6:** Top three master regulators upstream of dysregulated genes in GE1-HCC and GE2-HCC

Gene regulation in HCC compared with NT	HCC subtype	Master molecule name	Reached from input set	FDR	Median FC gene expression in GE1-HCC	Median FC gene expression in GE2-HCC
		TGM2	10	0.001	NC	NC
	GE1	MMP15	9	0.001	NC	NC
UP		E2F1	8	0.003	1.97	6.43
		CCNB2	225	0.001	NC	8.02
	GE2	APC/C	219	0.001	NC^a^	2.20^a^
		PKMYT1	232	0	NC	4.05
		SOCS3	51	0	− 2.03	− 1.95
	GE1	CD4	40	0	− 2.03	NC
DOWN		IL10	56	0	NC	NC
		TNF	147	0	NC	NC
	GE2	GRK5	96	0	NC	− 2.51
		IL10	134	0	NC	NC

“Reached from input set”—the number of genes from the input list present in the network of this master molecule.

*FC* fold change, *FDR* false discovery rate, *HCC* hepatocellular carcinoma, *NC* no change, *NT* non-tumourous

^a^FC is shown for APC subunit ANAPC11

The top regulatory molecules identified in the networks of genes downregulated in the GE1-HCC (115 input genes) and GE2-HCC (464 input genes) were related to reduced anti-tumour immune response and inflammation.

### Transcriptional regulators of gene networks in GE1-HCC and GE2-HCC

A bioinformatic search for potential transcription factor binding sites (TFBSs) using the MATCH™ tool and TRANSFAC® library of positional weight matrices integrated in the GeneXplain platform identified a number of transcription factors potentially involved in the coordinated regulation of gene expression in the GE1-HCC and GE2-HCC subgroups (Additional files 15–18). The majority of the top potential transcriptional regulators identified have already been found to play roles in various cancers, including HCC (Tables 7 and 8), thus demonstrating the validity of our in silico findings.

**Table 7:** Top* TFBSs enriched in genes upregulated in GE1-HCC

ID TRANSFAC	Gene symbol	Yes/no ratio	*P*	Involvement in cancer types other than HCC	Involvement in HCC
V$AR_03	*AR*	14.1	2.39E−02	Tumour promoting [23]	Tumour promoting [24]
V$TFIIA_Q6	*GTF2A1*/*GTF2A2*	8.5	1.25E−02	General enhancement of gene transcription [25]	–
V$PR_01	*PGR*	7.1	5.47E−02	Tumour promoting [26]	Unclear [27]
V$HES1_Q2	*HES1*	7.1	1.79E−02	Tumour promoting [28]	Unknown
V$ZNF219_01	*ZNF219*	4.4	3.10E−14	Unknown	Unknown
V$AREB6_03	*ZEB1*	4	4.24E−03	Tumour promoting [29]	Tumour promoting [30, 31]
V$GCNF_01	*NR6A1*	3.2	2.99E−02	Tumour promoting [32]	Unknown
V$MAZR_01	*PATZ1*	2.7	2.16E−50	Tumour promoting [33]	Unknown
V$PAX4_04	*PAX4*	2.7	3.70E−02	Cell proliferation/survival promoting or tumour suppressing in different contexts [34]	Unknown
V$UF1H3BETA_Q6	*UF1-HNF3B* (not further identified)	2.6	3.09E−74	–	–
V$CACBINDINGPROTEIN_Q6	CAC-binding protein (not further identified)	2.5	1.87E−26	–	–
V$MTF1_Q4	*MTF1*	2.5	3.30E−04	Tumour promoting [35]	Unknown

*HCC* hepatocellular carcinoma, *TFBS* transcription factor binding site

*TFBS with Yes/No ratio > 2.5 are included. “Yes” set—promoter sequences of the genes co-regulated in the tumours. “No” set—promoter sequences of the control genes. Yes/No ratio—ratio of the frequencies of TFBSs in the “Yes” gene set and “No” gene set. Yes/No ratio > 1 indicates overrepresentation of TFBS in the co-regulated genes and suggests potential involvement of the corresponding transcription factor in regulation of the observed expression changes

**Table 8:** Top* TFBSs enriched in genes upregulated in GE2-HCC

ID TRANSFAC	Gene symbol	Yes–no ratio	*P*	Involvement in cancer types other than HCC	Involvement in HCC
V$NKX61_01	*NKX6-1*	6.17	5.13E−02	Diagnostic marker [36]	Tumour promoting [37]
V$OCT_C	*POU2F1*	6.17	4.20 E−05	Tumour promoting [38]	Tumour promoting [39]
	*POU2F2*			Tumour promoting [40]	Unknown
V$OCT1_Q6	*POU2F1*	4.28	6.61 E−05	Tumour promoting [38]	Tumour promoting [39]
V$VJUN_01	*JUN*	5.29	1.88 E−03	Tumour promoting [41]	Predictive biomarker [42]
V$LHX3A_01	*LHX3*	4.85	1.97 E−02	Tumour promoting [43]	Unknown
V$IRF_Q6	*IRF1* through *IRF8*	4.7	4.96 E−03	Tumour suppressing [44]	Tumour suppressing [45]
V$E2F_03	*E2F1*	3.29	6.29 E−06	Tumour promoting [46]	Tumour promoting [47]
V$HSF2_01	*HSF2*	3.23	5.03 E−03	Suggested tumour inhibiting [48]	Unknown
V$SRF_Q4	*SRF*	2.94	7.27 E−02	Predictive marker [49]	Tumour promoting [50]
V$GABP_B	*GABPA*/ *GABPB1*	2.86	4.36 E−02	Suggested tumour promoting [51, 52]	Negative prognostic marker [53]
V$TFIIA_Q6	*GTf2A1*/ *GTf2A2*	2.82	2.67 E−02	General enhancement of transcription [25]	–
V$AFP1_Q6	*ZFHX3*	2.64	6.02 E−03	Tumour suppressing [54]	Unknown
V$PR_01	*PGR*	2.64	6.45 E−02	Tumour promoting [26]	Unclear [27]
V$CAAT_01	cellular and viral CCAAT-box	2.13	6.61 E−11	–	–
V$CP2_02	*TFCP2*	2.1	6.98 E−04	Tumour promoting [55]	Tumour promoting [55, 56]
V$NFY_01	*NFYA*, *NFYB*, *NFYC*	1.65	3.06 E−09	Prognostic marker [57, 58]	Unknown
V$CHX10_01	*VSX2*	1.61	4.73E−04	Diagnostic marker, suggested tumour inhibiting [59]	Unknown

“Yes” set—promoter sequences of the genes co-regulated in the tumours. “No” set—promoter sequences of the control genes. Yes/No ratio—ratio of the frequencies of TFBSs in the “Yes” gene set and “No” gene set. Yes/No ratio > 1 indicates overrepresentation of TFBS in the co-regulated genes and suggests potential involvement of the corresponding transcription factor in regulation of the observed expression changes

*HCC* hepatocellular carcinoma, *TFBS* transcription factor binding site

*TFBS with Yes/No Ratio > 2.5 or Yes/No Ratio > 1.5 and *P* < 0.001 are included

The results in GE1-HCC (Table 7, Additional file 15) focus at first on androgen receptor signalling (AR) (Yes/No ratio = 14.1), whereas its role in GE2-HCC seems to be low, as indicated by a Yes/No ratio of 1.1 (Additional file 16). Furthermore, expression data showed *AR* downregulation (mean FC = -3.22) in advanced GE2-HCC (Additional file 5).

Upregulated genes in GE1-HCC also showed enrichment in TFBSs for progesterone (*PGR*). Unlike *AR*, *PGR* binding sites enrichment was found also in GE2-HCC (Table 8). We also identified potential transcriptional factors with known oncogenic roles in HCC, e.g. ZEB1, or in other cancer types, e.g. HES1, NR6A1, PATZ1, PAX4 and MTF1, in GE1-HCC. A novel finding was the transcription factor ZNF219; its binding site showed a high Yes/No ratio of 4.4 and was found in promoters of more than half of upregulated genes in GE1-HCC.

In GE2-HCC, top potential transcriptional activators included transcription factors with reported tumour-promoting activity in HCC and/or other cancer types, e.g., NKX6-1, POU2F1/2, cJUN, E2F1, HSF2, SRF, TFCP2 and NFY (Table 8).

Some transcription factors seem to have an important role in activation of gene expression in both GE1-HCC and GE2-HCC. These were general transcription factors (GTF2A1/2) with a role in general enhancement of transcription and *PGR*, as mentioned above. A few transcription factors with high rank in one group (Tables 7 and 8) might also play a less significant role in the other group, where they were ranked lower (Additional files 15 and 16). This phenomenon relates to *ZEB1* and *PATZ1*, which binding sites were highly presented in genes upregulated in GE1-HCC but less so in GE2-HCC. By contrast, the rank of E2F1, GABP and TFCP2 in regulation of gene networks in liver carcinomas strongly increased from GE1-HCC to GE2-HCC.

## Discussion

As described by Edmondson and Steiner in 1954, HCC includes a heterogeneous group of carcinomas [11]. Morphological dedifferentiation is accompanied by increased genomic instability, in particular chromosomal instability, and a worse prognosis [6–8]. In this study, we demonstrated that molecular tumour typing based on gene expression profiling correlated to histological grade and chromosomal instability—defining two main types of HCC—and may be used for detection of molecular markers to improve HCC therapy options.

### DNA level/chromosomal instability

Previously reported HCC chromosomal changes include gains of 1q, 6p, 8q, 17q and 20q and losses of 1p, 4q, 6q, 8p and 13q. Furthermore, 4q and 13 q deletions have been repeatedly correlated to HCC dedifferentiation [17, 60–62]. We also observed these characteristic aberrations in our set of hepatic malignancies. Amplification of genomic material could contribute to ectopic expression of genes with essential roles in cell growth, survival, and proliferation (Table 3). Notably, while gains of genetic material were observed in both the GE1-HCC and GE2-HCC subgroups, losses were observed mostly in the GE2-HCC subgroup (i.e., high-grade tumours). We assume that the loss of genetic material observed in GE2-HCCs is a result of severely dysregulated control of mitotic mechanisms and chromosomal missegregation resulting from progressive malignant derailment. Interestingly, recurrent gains at 7q, 8q, 11p, 19p, and 19q were also observed in the adjacent NT tissues from both HCC sample groups and most likely represent initial cancer-predisposing events.

Similar to the results in our previous study of childhood HCCs [17] we observed recurrent gain of chromosome 19 in GE1-HCC (100% frequency) and GE2-HCC (60% frequency) as well as in 50% of adjacent NT tissues. This finding may indicate a particular role for this chromosomal aberration in the development of liver carcinogenesis.

### RNA level

Overall, gene expression analysis revealed two main HCC groups: GE1-HCC and GE2-HCC, with GE1-HCC closer to NT than to GE2-HCC—the latter typically having a higher histological grade and higher level of chromosomal instability (as described above). There were a number of dysregulated genes in GE1-HCC and GE2-HCC, suggesting their essential role in the initiation and development of liver carcinogenesis. By contrast, other genes that were only dysregulated in GE2-HCC indicated their specific roles in cancer progression.

#### Genes upregulated in GE1-HCC and GE2-HCC

The genes significantly dysregulated in GE1-HCC and GE2-HCC revealed changes in the same direction; most of the dysregulation was more pronounced in GE2-HCC. The only exception was the long non-coding RNA (lncRNA) *H19*, which is frequently reported as dysregulated in cancer including HCC, sometimes up- and sometimes downregulated [63, 64]. In our samples, *H19* was highly expressed in NT, downregulated in GE1-HCC and upregulated in GE2-HCC. These findings support the notion about the essential but context-specific role of *H19* during all steps of liver carcinogenesis. Most of the genes upregulated in both GE1-HCC and GE-2 HCC have been reported to exert an oncogenic action. For example, the lncRNA *HNF1A-AS1* is functionally involved in various carcinomas including HCC [65–69]. Another example is *MDK* (encodes a secreted growth factor): its upregulation correlated with a worse prognosis and chemotherapy resistance in diverse malignant tumours including HCC [70–72]. MDK-targeted molecular therapy approaches are under investigation [73]. Other genes with increased expression in GE2-HCC compared with GE1-HCC included transcription factor *E2F1,* with a crucial role in cell cycle regulation, *BIRC5* (survivin), an inhibitor of apoptosis, and helicase *RECQL4,* essential for maintaining genomic stability. The overexpression of these genes is critical for the development of various malignancies (including HCC) [47, 74–79]. Moreover, there have been reported direct interactions of E2F1 and retinoblastoma protein (RB) with the promoter of *RECQL4* in prostate cancer cells [77] and RECQL4 with BIRC5 in breast cancer cells [78]. These data suggest a ↑E2F1 → ↑RECQL4 → ↑BIRC5 axis in oncogenic derailment. A new finding in our study was upregulation of *C1orf35*, a novel potential oncogene [80]. To our knowledge, our study is the first to show its overexpression in solid tumours. Notably, we found the genomic loci coding for *H19* (11p15.5), *RECQL4* (8q24.3), *E2F1* (20q11.2), *BIRC5* (17q25.3) and *C1orf35* (1q42.13) to be frequently amplified in both GE1-HCC and GE2-HCC. Furthermore, loci 11p15.5 and 8q24.3 were frequently amplified in NT tissues. These findings suggest the role of gene amplification in the overexpression of these genes.

#### Genes downregulated in GE1-HCC and GE2-HCC

The genes repressed in both GE1-HCC and GE2-HCC included those with supposed tumour-inhibiting activity, e.g. *CXCL14*, *CCBE1, IGFBP1*, *RND3, BMP10* and *COLEC10*, which encode proteins involved in extracellular matrix remodelling, migration and the immune response. Their decreased expression in various cancers has been reported [81–86].

#### Genes upregulated in GE1-HCC

A few genes specifically upregulated in GE1-HCC have been found to promote carcinogenesis of various cancer types when overexpressed. Some examples are *CACNA1H*, *DBP*, *B3GnT8*, *ASAP3* and *CYHR1,* with *CACNA1H* and *DBP* linked to chemoresistance [87, 88]. One exception is *DNM3*, a gene that encodes a protein with growth-inhibiting function in HCC cells [89].

#### Genes upregulated in GE2-HCC: cell proliferation

Most of the genes strongly upregulated only in the GE2-HCC subgroup (Additional files 5 and 6) encode proteins involved in the cell cycle and its regulation. Many of these genes have previously been linked to advanced stages of different cancer types including HCC. These genes enclose *KIFC1* [90, 91], *GPC3* [92], *PEG10* [93–95]*,* and *UBE2C* [96–98].

In GE2-HCC, there was marked upregulation (up to 17 fold) in the expression of several small non-coding RNAs—*SNAR-A3*, *SNAR*-*B2*, *SNAR*-*H*, *SNAR-I*, *SNAR*-*D* and *SNAR*-*G2*—located on 19q13.33, a genomic region that showed recurrent gain in HCC and NT samples. An in vivo study showed that the *SNAR* family members are associated with NF90, a double-stranded RNA (dsRNA) binding protein implicated in transcriptional and translational control [99] and suggested to be involved in viral replication, e.g. hepatitis C virus [100]. *ILF3*, which encodes the NF90 protein, was also upregulated in GE2-HCC and located on the amplified 19p13.2 region. A recent study reported ectopic expression of some *SNAR* family members in HCC patients. This dysregulation promoted invasion and migration in HCC cells [101].

#### Genes upregulated in GE2-HCC: nuclear size and transport

The distinct feature of tumour cells is an enlarged nucleus, although the molecular mechanisms that regulate nuclear size in normal and cancer cells remains unknown. We found that the expression of two major nuclear transport receptor genes, importin alpha 1—also known as karyopherin alpha 2 (*KPNA2*) —and exportin (*XPOT*), were significantly increased specifically in GE2-HCC. These changes could lead to profound dysregulation of nuclear-cytoplasmic transport [102]. KPNA2 is the most abundant nuclear import receptor for proteins. An excess of this protein directly increased the nuclear size in a *Xenopus* model [103]. It contributes to enhanced nuclear entry of structural proteins necessary for building up the nuclear envelope. The signalling pathways are also dependent on the nuclear transport system, namely import of activated transcription factors and signalling proteins and export of target gene products. Thus, *KPNA2* overexpression might strongly deregulate gene transcription associated with increased proliferation and survival in GE2 carcinomas. Increased *KPNA2* expression has been found in HCC patients, and its overexpression correlated with the expression of essential mitotic proteins CCNB2 and CDK1 in HCC cells [104]. These findings are consistent with our study. Similar results have been reported for exportin-t (encoded by *XPOT*), a nuclear export receptor responsible for most export of mature tRNA from the nucleus to cytoplasm. The enhanced export of mature tRNA could hasten protein synthesis in the cytoplasm. A study revealed *XPOT* upregulation in HCC tumour tissues, poor outcome for patients with the high expression of the protein and association of *XPOT* expression with expression of molecules that regulate the cell cycle (i.e. several cyclins and cyclin-dependent kinases) in HCC cells [105].

#### Genes upregulated in GE2-HCC: chromosomal instability

GE2-HCC showed a high level of chromosomal instability, with large genomic losses and aneuploidy. This dysfunction might be linked to overexpression of genes involved in DNA repair, e.g. *HJURP* and *PARP1*, and in chromosome segregation and spindle checkpoint function, e.g. *CDC20*, *ANAPC1*, *ANAPC11*, *TTK*, *BUB1B*, *MAD2L1*, *KIFC1*, *PTTG1* and *UBE2C*. *HJURP* encodes a protein involved in the repair of double-stranded DNA breaks. Its overexpression leads to mitotic defects in human cells [106]. HJURP has already been shown to be an independent prognostic marker in HCC [107, 108]. PARP1 is involved in microhomology-mediated end joining (MMEJ), an error-prone DNA repair pathway. PARP1 is aberrantly expressed in different cancers, where its overexpression rather than underexpression is associated with genomic instability, likely due to accumulation of errors through increased MMEJ [109]. Higher PARP1 expression has been correlated to a higher stage in HCC [110]. PARP1 inhibitors are already in clinical use to suppress various tumours [109]. We also observed high expression of some other genes involved in the spindle checkpoint mechanism and chromosome segregation, e.g. *CDC20*, *MAD2L1* and TTK. Their overexpression, as opposed to mutation or underexpression, has been found in cancer cells with genomic instability and mitotic spindle defects [111], and has also been reported in HCC, where it was linked to low patient survival [112, 113].

### Master regulators in GE1-HCC

Master regulators identified for upregulated gene networks in GE1-HCC included membrane-type metalloproteinases (MT-MMPs), TGM2 and E2F1. TGM2 is a cell-surface protein involved in cell binding to fibronectin. Fibronectin is a reactive extracellular matrix component implicated in cell adhesion, migration and signalling in the liver [114]. Abnormal fibronectin accumulation in the liver due to sustained injury or disease has been suggested to elicit aberrant cell signalling that promotes further tissue damage and contributes to tumour genesis [114, 115]. E2F1 plays a critical role in the control of cellular proliferation and can activate genes that promote cell proliferation or apoptosis. Pro-survival signalling might abrogate the pro-apoptotic capacity of E2F1 and thus favour E2F1-mediated cell proliferation. MMP15 (associated with the plasma membrane) and TGM2, besides their involvement in invasion and metastasis, can convey cell signalling to enhance cell survival [116, 117]. Therefore, MT-MMPs, TGM2 and E2F1 might be conjoined by pathological processes in the liver to trigger inappropriate cell proliferation and tumour development. In our study, there was a nearly twofold increase in *E2F1* in GE1-HCC. We discuss its pivotal role in the process of liver transformation below.

### Transcriptional regulators in GE1-HCC

A bioinformatics search identified AR as an important transcription factor involved in upregulation of genes in GE1-HCC, with *BIRC5* (an apoptosis inhibitor) and *C1orf35* (a transcript with transforming activity) as its possible target genes. Androgen-dependent and androgen-independent AR activation has been reported in hepatocarcinogenesis [24]. However, anti-androgenic treatment did not benefit male patients with advanced HCC [118]. Consistent with this observation, our analysis showed that in GE2-HCC, *AR* gene expression and its role in gene transcription (Yes/No ratio in regulated genes) were significantly reduced compared with GE1-HCC. Information about PGR role in HCC is rare [26]. The results of our study support the notion that PGR may be implicated in HCC carcinogenesis at early and advanced stages of the disease. Understanding the roles of AR and PGR in HCC is important because AR and PGR inhibitors are already in clinical use [24, 27]. A number of other potential transcriptional regulators identified in GE1-HCC—e.g. ZEB1, HES1, NR6A1, PATZ1, PAX4 and MTF1—have a reported oncogenic role in cancer types other than HCC [28–35]. Our results implicate them in hepatocarcinogenesis too. Transcription factor ZNF219, whose potential binding sites are found in several genes with an obvious role in carcinogenesis, i.e. *E2F1*, *MDK*, *REQL4* and *C1orf35*, has emerged as a new potential transcriptional regulator in GE1-HCC.

### Master regulators in GE2-HCC

The most significant master regulators identified for upregulated gene networks in GE2-HCC are important regulators of the cell cycle, namely the G2/M transition and mitotic spindle formation. The kinase PKMYT1 inhibits the G2/M transition, allowing time for DNA damage recovery. It is overexpressed rather than underexpressed in HCC, and exerts oncogenic activity in HCC cells [119]. Targeted inhibition of PKMYT1 activity would shorten the time between checkpoint abrogation and mitotic entry and, therefore, should preferably damage cancer cells with an already defective G1 checkpoint mechanism. Efforts have been made to develop small molecule inhibitors of PKMYT1 as a promising anti-cancer therapy approach [120]. CCNB2 overexpression hyperactivates the centrosomal kinases AURKA and PLK1, leading to accelerated centrosome separation, lagging chromosomes and aneuploidy [121]. The APC/C complex bound to the regulatory protein CDC20 initiates anaphase entry for chromosome separation, and CDC20 is a critical molecule in the spindle assembly checkpoint mechanism. The ubiquitin-conjugating enzyme UBE2C collaborates with the APC/C–CDC20 complex in the removal of mitotic regulators and thereby contributes to cell cycle progression. Increased CDC20 expression has been associated with defective spindle formation and progression of multiple tumours, including HCC [122]. UBE2C overexpression also leads to chromosomal missegregation and tumour formation [123]. Nath et al. [124] linked UBE2C overexpression and chromosomal instability in cancer cells to excess E2F1, which recruits the CDC20–APC/C to upregulate *UBE2C* transcription. This phenomenon highlights the role of deregulated RB–E2F1 pathway in premature anaphase, chromosomal abnormalities and aneuploidy beyond its well-documented role in G1/S progression. All aforementioned genes, i.e. *CCNB2*, *PKMYT*, some components of the APC/C complex (e.g. *ANAPC1* and *ANAPC11*), *CDC20*, *UBE2C*, *E2F1* and many others involved in progression and control of G2/M phase, were strongly upregulated in GE2-HCC. They might represent candidates for targeted therapy in advanced HCCs with high-proliferation signature.

### Transcriptional regulators in GE2-HCC

Promoter analysis of genes upregulated in GE2-HCC suggests the activation of transcription factor NKX6.1. A few upregulated genes with potential TFBSs for NKX6-1 in their promoters are critical genes essential for cell proliferation (*CDK1* and two replication-dependent histones), growth (*KPNA2* and *XPOT*) and motility (*ROBO1*) and might mediate the oncogenic effects of NKX6-1. NKX6-1 overexpression in HCC is associated with progressive features and an unfavourable prognosis [37].

Transcription factor POU2F1 might be pro-oncogenic in multiple contexts. It has been implicated in cell proliferation, immune modulation, oxidative and cytotoxic stress resistance, metabolic regulation, stem cell identity and a poised transcription state [38, 125]. It has been hypothesised to integrate multiple signal inputs through diverse posttranslational modifications and interaction with various partners to direct changes in gene transcription in general, by opening chromatin, and in cell-specific contexts. In prostate cancer, POU2F1 is a co-regulator of AR, leading to AR hypersensitivity and driving androgen-independent cancer progression [126]. A similar phenomenon might occur in liver carcinogenesis. Accumulation of DNA damage and cell stress during the disease progression might activate POU2F1, leading, in turn, to changes of gene transcription induced by other transcription factors through modifying their interaction with ligands and/or binding to DNA. Enhanced POU2F1 activity has been described in different epithelial malignancies as a result of focal amplification, increased mRNA, augmented protein level and enhanced activity [38]. We found amplification of the 1q24.2 locus, which encodes *POU2F1*, in more than 80% of all HCCs, but its transcriptional level was not changed. These data suggest the involvement of other mechanisms in the enhancement of its transcriptional activity. POU2F1 has already emerged as a prognostic marker and a potential therapeutic target in several types of cancer including HCC [39]. The fact that POU2F1 is important for the cell response to genotoxic and oxidative stress, but is not critical under standard conditions, makes its inhibition an attractive anti-cancer therapeutic approach. One tactic is targeting the POU2F1-binding DNA sequence by pyrrole-imidazole polyamides, which has been successfully implemented in mice [126].

The second highest transcriptional regulator in GE2-HCC was the proto-oncogene JUN, overexpression of which has been shown to reprogramme oestrogen receptor signalling in breast cancer cells and confer them resistance to tamoxifen [41]. JUN overexpression in HCC has been linked to sorafenib resistance [42].

Another significant transcription factor was E2F1, the expression of which was also strongly increased in GE2-HCC. As discussed in the section about master regulator molecules, the E2F family plays a crucial role in controlling the cell cycle and the action of tumour suppressor proteins [74]. When overexpressed, it can be responsible for promoting the cell cycle as well as genomic instability [124]. Sustained activation of E2F1 and its target genes might be induced by impaired regulatory mechanisms and aberrant signalling due to chronic liver disorders or injury or by DNA tumour virus oncoproteins, e.g. the hepatitis B virus X protein known to target the Rb–E2F1 pathway [127]. Subtle yet significant gains in *E2F1* and *E2F3* copy number rather than mutations have been found in advanced HCC, and a direct, cell-autonomous role for *E2f* activators in driving HCC in mice has been demonstrated [47]. In our HCC cohort, the genetic locus 20q11.2, which encodes *E2F1*, was recurrently amplified in tumours, *E2F1* expression was moderately and strongly upregulated in GE1-HCC and GE2-HCC, respectively.

To achieve growth regulation, E2F1 requires partners. One of these is NFY [128]. More than a half of the genes upregulated in GE2-HCC with a high proliferation signature were enriched in CCAAT box and NFY binding motif for CCAAT-binding transcription factor NFY. This finding agrees with studies of other cancers where CCAAT boxes were over-represented and bound to NFY in variety of upregulated genes [57]. NFY is an essential regulator of cell cycle progression. It activates transcription of numerous genes that regulate cell cycle, including *E2F1*. It also has a direct non-transcriptional role in the overall efficiency of DNA replication. NFY increases cell type–specific gene expression by promoting chromatin accessibility for cell type–specific master transcription factors [129]. Therefore, NFY activation might contribute to a broad augmentation of gene expression changes, first of all, *E2F1* overexpression, associated with cancer progression in GE2-HCC. Considerable efforts have being made to find compounds that specifically inhibit NFY activity in cancer cells [57].

The transcription factor GABP is a negative prognostic biomarker in HCC and inhibits HCC cell migration and invasion [53]. On the other hand, GABP strongly binds and activates a mutant promoter of the telomerase reverse transcriptase gene (*TERT*), reactivating its expression and thereby increasing the replicative potential of tumour cells [51]. Two specific non-coding mutations in the *TERT* promoter required for the activation of *TERT* transcription by GABP occur with high frequency in aggressive cancer types. In one study, these mutations were found in 47% of HCCs [130]. In our study, the promoter analysis identified GABP as potential important transcriptional regulator in GE2-HCC, but we did not find a significant difference in *TERT* transcription among GE1-HCC, GE2-HCC and NT samples.

Two additional transcriptional regulators upregulated in GE2-HCC were *SRF* and *TFCP2*. Increased SRF and TFCP2 levels accelerate tumour cell migration and invasion and are linked to cancer progression [49, 55] and to high-grade HCC [50, 56]. TFCP2 is involved in regulation of cell proliferation, invasion, angiogenesis, metastasis and chemoresistance in HCC [56]. Small molecular inhibitors of TCPF2 have emerged as promising potent and effective therapeutics for HCC [131].

### Candidate molecules for targeted anti-tumour therapy in GE1-HCC and GE2-HCC

As described above, the GE1-HCC and GE2-HCC subgroups were determined based on specific changes in histological, DNA and RNA levels that reflect their different biology. As a result, we uncovered many potentially valuable targets for anti-cancer therapy, as already discussed earlier and listed in Table 9.

**Table 9:** Candidate molecules for targeted anti-tumour therapy in GE1-HCC and GE2-HCC

Analyses predicting the candidate gene	Target molecule in GE1-HCC	Target molecule in GE2-HCC	Selectivity suggested for cancer cells	References to targeted therapy approaches under investigation/development
GE, MR, TR	E2F1	E2F1		[132]
GE	MDK	MDK	Yes	[73]
GE	BIRC5	BIRC5	Yes	[133]
GE	GPC3	GPC3	Yes	[92]
GE	–	KIFC1	Yes	[134]
GE	–	PARP1	Yes	[109]
GE, MR	–	PKMYT1	Yes	[119, 120]
GE		PEG10		[95]
MR, GE	SOCS3	–		[135]
MR	IL10	IL10	Yes	[136]
MR	–	TNF		[137]
TR	AR	–		[24]
TR	PGR	PGR		[27]
TR	–	POU2F1	Yes	[126]
TR	–	NFY		[57]
TR	TFCP2	TFCP2		[131]

*GE* gene expression, *HCC* hepatocellular carcinoma, *MR* master regulator, *TR* transcriptional regulator

Our findings point to a demand for differentiated approaches for treatment of HCC with low and high proliferation. Whereas E2F1, MDK, BIRC5, IL10, PGR, C1orf35 and TFCP2 (among others) seem to represent promising targets for intervention in all HCCs, therapy directed against KIFC1, PAFAH1B3, PKMYT1, PEG10, PARP1, POU2F1, NFY or TNF might be effective specifically in advanced GE2-HCC. Conversely, anti-androgen therapy, anti-ZNF219 and SOSC3-peptidomimetics may have a therapeutic potential for HCCs with low proliferation signature.

## Conclusion

The findings from this study, in accordance with abundant previously published data, argue for a pivotal role of dysregulation of the E2F1 pathway in liver carcinogenesis. This dysfunction, due to diverse pathological processes in the liver, is capable of initiating both inappropriate cell proliferation and chromosomal instability.

**Fig. 4:**
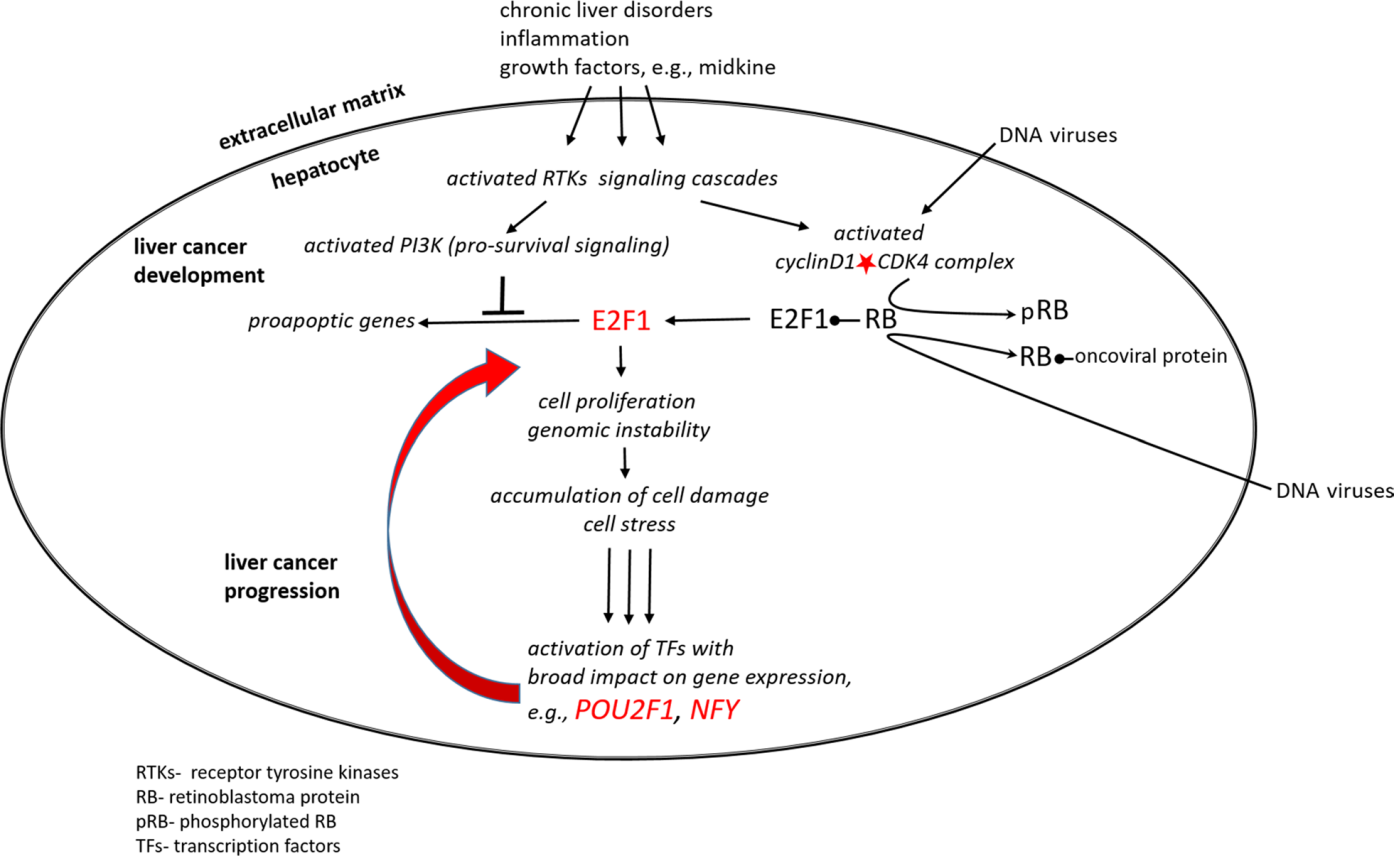
Proposed scheme for key steps in liver cancer development and progression

A dedifferentiation switch manifested by strong propagation of gene expression changes and genomic instability might be linked to turning on transcriptional co-regulators, e.g. POU2F1 (OCT1) and NFY, as a response to accumulating cell stress during malignant development (Fig. 4).

Activation of transcriptional co-activators might explain, at least in part, the insensitivity of advanced HCC to anti-androgen treatment.

Our findings highlight the demand for different approaches to treat HCC entities that exhibit low or high proliferation signatures and provide new, promising candidates for developing targeted HCC therapy.

## Supplementary Information

**Additional file 1**. Clinicopathological data of the patients with liver carcinoma used in the study.

**Additional file 2**. Differentially expressed probe sets in hepatocellular carcinoma (HCC) versus non-tumourous (NT) tissues. Unpaired Welch’s t-test was performed using gene expression microarray data from 23 HCC and 17 NT samples. The probe sets differentially expressed in the HCC group (n = 23) versus the NT group (n = 17) showing a mean absolute fold change (FC) ≥ 2, with a false discovery rate (FDR) ≤ 0.05, were selected and used for hierarchical cluster analysis and principal component analysis.

**Additional file 3**. Two subtypes of hepatocellular carcinoma (HCC), with distinctive gene expression patterns, were defined by hierarchical cluster analysis. Gene expression values from 23 HCC samples of various histological grades and 17 surrounding non-tumourous (NT) tissues were analysed. For the analysis, 458 probe sets differently expressed in the HCC samples versus the NT tissues with a mean absolute ratio ≥ 2 and false discovery rate ≤ 0.05 (Additional file 2) were used. The scale in the top right shows the colour codes representing gene expression values (Log2) – green for low expression and red for high expression.

**Additional file 4**. List of differentially expressed probe sets in GE1-hepatocellular carcinoma (HCC) versus non-tumourous (NT) tissues. Unpaired Welch’s t-test was performed using gene expression microarray data from GE1-HCC (n=10) and NT (n=17) samples. The probe sets demonstrating expression changes in the GE1-HCC group versus the NT group with a mean absolute fold change (FC) ≥ 2 and P ≤ 0.01 were selected and used for further bioinformatic analyses. 

**Additional file 5**. List of differentially expressed probe sets in 13 GE2-hepatocellular carcinoma (HCC) versus 17 non-tumourous (NT) tissues. Unpaired Welch’s t-test was performed using gene expression microarray data from GE2-HCC (n=13) and NT (n=17) samples. The probe sets demonstrating expression change in the GE2-HCC group versus the NT group with a mean absolute fold change (FC) ≥ 2 and false discovery rate (FDR) ≤ 0.05 were selected and used for further bioinformatics analyses.

**Additional file 6**. List of differentially expressed probe sets in 13 GE2-hepatocellular carcinoma (HCC) versus 10 GE1-HCC samples. Unpaired Welch’s t-test was performed using gene expression microarray data from GE2-HCC (n=13) and GE1-HCC (n=10) samples. The probe sets that expression change in the GE2-HCC group versus the GE1-HCC group showed mean absolute fold change (FC) ≥ 2 and false discovery rate (FDR) ≤ 0.05 were selected.

**Additional file 7**. Percentage of aberrations shared by multiple samples in the set of hepatocarcinoma (HCC) tissues. Genomic DNA isolated from HCC samples (n=22) was analysed using high resolution, microarray-based, comparative genomic hybridisation (aCGH). Human male genomic DNA (Promega) was used as a reference.

**Additional file 8**. Percentage of aberrations shared by multiple samples in the set of non-tumorous (NT) tissues. Genomic DNA isolated from NT samples (n=13) was analysed using high resolution, microarray-based, comparative genomic hybridisation (aCGH). Human male genomic DNA (Promega) was used as a reference.

**Additional file 9**. Percentage of aberrations shared by multiple samples in the set of hepatocarcinoma (HCC) of GE1 group. Genomic DNA isolated from GE1-HCC samples (n=10) was analysed using high resolution, microarray-based, comparative genomic hybridisation (aCGH). Human male genomic DNA (Promega) was used as a reference.

**Additional file 10**. Percentage of aberrations shared by multiple samples in the set of hepatocarcinoma (HCC) of GE2 group. Genomic DNA isolated from GE2-HCC samples (n=12) was analysed using high resolution, microarray-based, comparative genomic hybridisation (aCGH). Human male genomic DNA (Promega) was used as a reference.

**Additional file 11**. Master regulator molecules of genes upregulated in GE1-hepatocellular carcinoma (HCC). Significantly up regulated genes in GE1-HCC (n=10) versus NT (n=17) samples with mean fold change (FC) ≥ 2 and P ≤ 0.01 were identified (Additional file 4) and used as an input gene list for bioinformatics analyses. Master regulator molecules in the signal transduction pathways, at a distance of up to 10 steps upstream of the dysregulated genes, were identified using the curated database of pathways and protein interactions TRANSPATH®.

**Additional file 12**. Master regulator molecules of genes upregulated in GE2-hepatocellular carcinoma (HCC). Significantly up regulated genes in GE2-HCC (n=13) versus NT (n=17) samples with mean fold change (FC) ≥ 2 and false discovery rate (FDR) ≤ 0.05 were identified (Additional file 5) and used as an input gene list for bioinformatics analyses. Master regulator molecules in the signal transduction pathways, at a distance of up to 10 steps upstream of the dysregulated genes, were identified using the curated database of pathways and protein interactions TRANSPATH®.

**Additional file 13**. Master regulator molecules of genes downregulated in GE1-hepatocellular carcinoma (HCC). Significantly down regulated genes in GE1-HCC (n=10) versus NT (n=17) samples with mean fold change (FC) ≤ -2 and P ≤ 0.01 were identified (Additional file 4) and used as an input gene list for bioinformatics analyses. Master regulator molecules in the signal transduction pathways, at a distance of up to 10 steps upstream of the dysregulated genes, were identified using the curated database of pathways and protein interactions TRANSPATH®.

**Additional file 14**. Master regulator molecules of genes downregulated in GE2-hepatocellular carcinoma (HCC). Significantly down regulated genes in GE2-HCC (n=13) versus NT (n=17) samples with mean fold change (FC) ≤ -2 and false discovery rate (FDR) ≤ 0.05 were identified (Additional file 5) and used as an input gene list for bioinformatics analyses. Master regulator molecules in the signal transduction pathways, at a distance of up to 10 steps upstream of the dysregulated genes, were identified using the curated database of pathways and protein interactions TRANSPATH®.

**Additional file 15**. Lists of overrepresented transcription factor binding sites (TFBSs) in genes upregulated in GE1-hepatocellular carcinoma (HCC). Significantly upregulated genes in GE1-HCC (n=10) versus NT (n=17) samples with mean fold change (FC) ≥ 2 and P ≤ 0.01 (Additional file 4) were used as an input gene list for bioinformatics analyses. Promoter sequences of the co-regulated in tumours genes (“Yes” set) and the control gene set (“No” set) were searched for potential transcription factor binding sites (TFBSs) using the MATCH^TM^ tool and TRANSFAC® library of positional weight matrices. The frequencies of TFBSs in the “Yes” gene set and “No” gene set were identified, and the ratio of “Yes” versus “No” was calculated. Yes/No ratio > 1 indicates overrepresentation of TFBSs in the co-regulated genes and suggests potential involvement of the corresponding transcription factor in regulation of the observed expression changes.

**Additional file 16**. Lists of overrepresented transcription factor binding sites (TFBSs) in genes upregulated in GE2-hepatocellular carcinoma (HCC). Significantly up regulated genes in GE2-HCC (n=13) versus NT (n=17) samples with mean fold change (FC) ≥ 2 and false discovery rate (FDR) ≤ 0.05 (Additional file 5) were used as an input gene list for bioinformatics analyses. Promoter sequences of the co-regulated in tumours genes (“Yes” set) and the control gene set (“No” set) were searched for potential transcription factor binding sites (TFBSs) using the MATCH^TM^ tool and TRANSFAC® library of positional weight matrices. The frequencies of TFBSs in the “Yes” gene set and “No” gene set were identified, and the ratio of “Yes” versus “No” was calculated. Yes/No ratio > 1 indicates overrepresentation of TFBSs in the co-regulated genes and suggests potential involvement of the corresponding transcription factor in regulation of the observed expression changes.

**Additional file 17**. Lists of overrepresented transcription factor binding sites (TFBSs) in genes downregulated in GE1-hepatocellular carcinoma (HCC). Significantly down regulated genes in GE1-HCC (n=10) versus NT (n=17) samples with mean fold change (FC) ≤ -2 and P ≤ 0.01 (Additional file 4) were used as an input gene list for bioinformatics analyses. Promoter sequences of the co-regulated in tumours genes (“Yes” set) and the control gene set (“No” set) were searched for potential transcription factor binding sites (TFBSs) using the MATCH^TM^ tool and TRANSFAC® library of positional weight matrices. The frequencies of TFBSs in the “Yes” gene set and “No” gene set were identified, and the ratio of “Yes” versus “No” was calculated. Yes/No ratio > 1 indicates overrepresentation of TFBSs in the co-regulated genes and suggests potential involvement of the corresponding transcription factor in regulation of the observed expression changes.

**Additional file 18**. Lists of overrepresented transcription factor binding sites (TFBSs) in genes downregulated in GE2-hepatocellular carcinoma (HCC). Significantly down regulated genes in GE2-HCC (n=13) versus NT (n=17) samples with mean fold change (FC) ≤ -2 and false discovery rate (FDR) ≤ 0.05 (Additional file 5) were used as an input gene list for bioinformatics analyses. Promoter sequences of the co-regulated in tumours genes (“Yes” set) and the control gene set (“No” set) were searched for potential transcription factor binding sites (TFBSs) using the MATCH^TM^ tool and TRANSFAC® library of positional weight matrices. The frequencies of TFBSs in the “Yes” gene set and “No” gene set were identified, and the ratio of “Yes” versus “No” was calculated. Yes/No ratio > 1 indicates overrepresentation of TFBSs in the co-regulated genes and suggests potential involvement of the corresponding transcription factor in regulation of the observed expression changes.

## Data Availability

The transcriptomic dataset generated during the current study and the detailed protocols used have been deposited in the ArrayExpress database at EMBL-EBI (www.ebi.ac.uk/arrayexpress) under accession number E-MTAB-8887. The genomic dataset generated during the current study and the detailed protocols used are deposited in the ArrayExpress database at EMBL-EBI (www.ebi.ac.uk/arrayexpress) under accession number E-MTAB-8886.

## Abbreviations

HCC: Hepatocellular carcinoma
GE1/2: Gene expression sub-group 1/2
NT: Non-tumourous liver tissue
aCGH: Array-based comparative genomic hybridisation
PCA: Principal component analysis
HCA: Hierarchical clustering analysis
FDR: False discovery rate
FC: Fold change
Chr: Chromosome
TFBS: Transcription factor binding site
