# Supplementary figures and images for: Gene networks and transcriptional regulators associated with liver cancer development and progression
Source: BMC Med Genomics. 2021 Feb 4;14:41. doi: 10.1186/s12920-021-00883-5 (PMC7863452; doi:10.1186/s12920-021-00883-5)

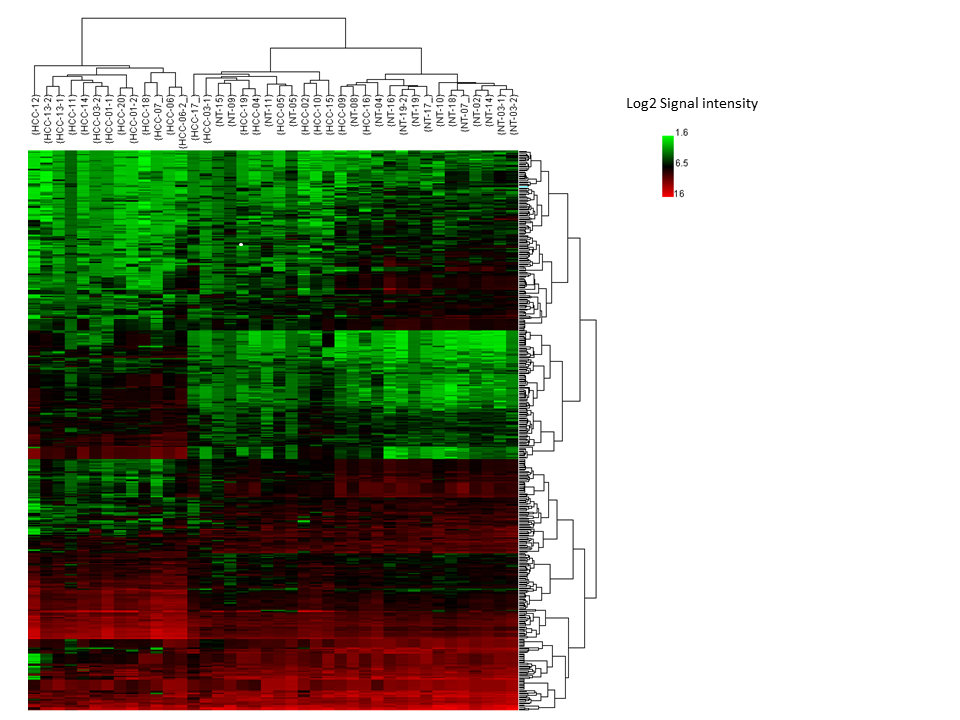

Supplement: Supplementary file 3 — Additional file 3. Two subtypes of hepatocellular carcinoma (HCC), with distinctive gene expression patterns, were defined by hierarchical cluster analysis. Gene expression values from 23 HCC samples of various histological grades and 17 surrounding non-tumourous (NT) tissues were analysed. For the analysis, 458 probe sets differently expressed in the HCC samples versus the NT tissues with a mean absolute ratio ≥ 2 and false discovery rate ≤ 0.05 (Additional file 2) were used. The scale in the top right shows the colour codes representing gene expression values (Log2) – green for low expression and red for high expression. [file 12920_2021_883_MOESM3_ESM.png]

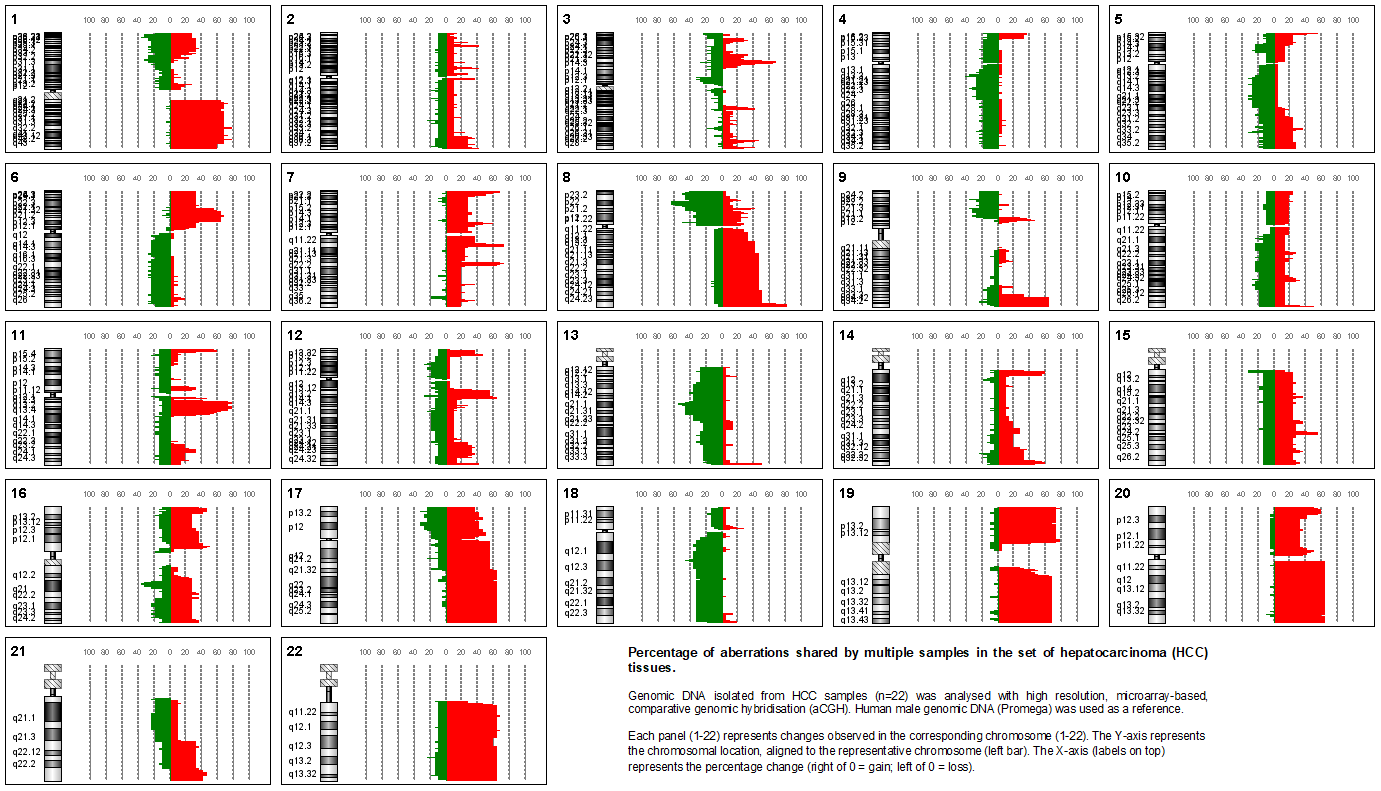

Supplement: Supplementary file 7 — Additional file 7. Percentage of aberrations shared by multiple samples in the set of hepatocarcinoma (HCC) tissues. Genomic DNA isolated from HCC samples (n=22) was analysed using high resolution, microarray-based, comparative genomic hybridisation (aCGH). Human male genomic DNA (Promega) was used as a reference. [file 12920_2021_883_MOESM7_ESM.tif]

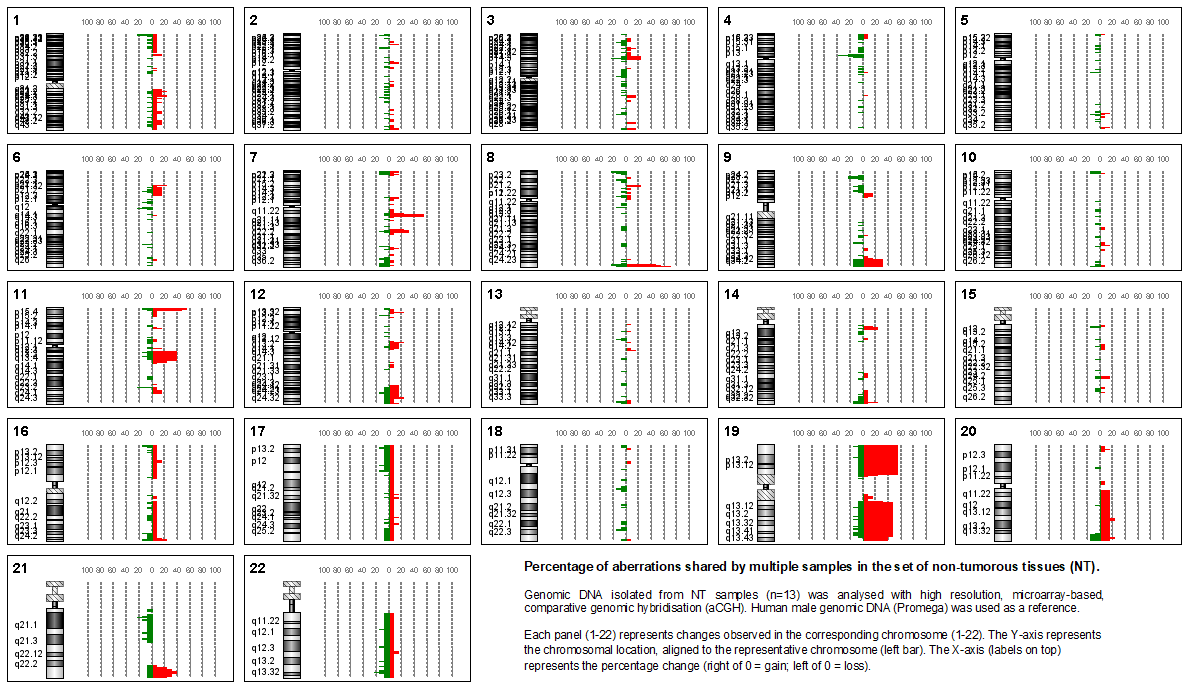

Supplement: Supplementary file 8 — Additional file 8. Percentage of aberrations shared by multiple samples in the set of non-tumorous (NT) tissues. Genomic DNA isolated from NT samples (n=13) was analysed using high resolution, microarray-based, comparative genomic hybridisation (aCGH). Human male genomic DNA (Promega) was used as a reference. [file 12920_2021_883_MOESM8_ESM.tif]

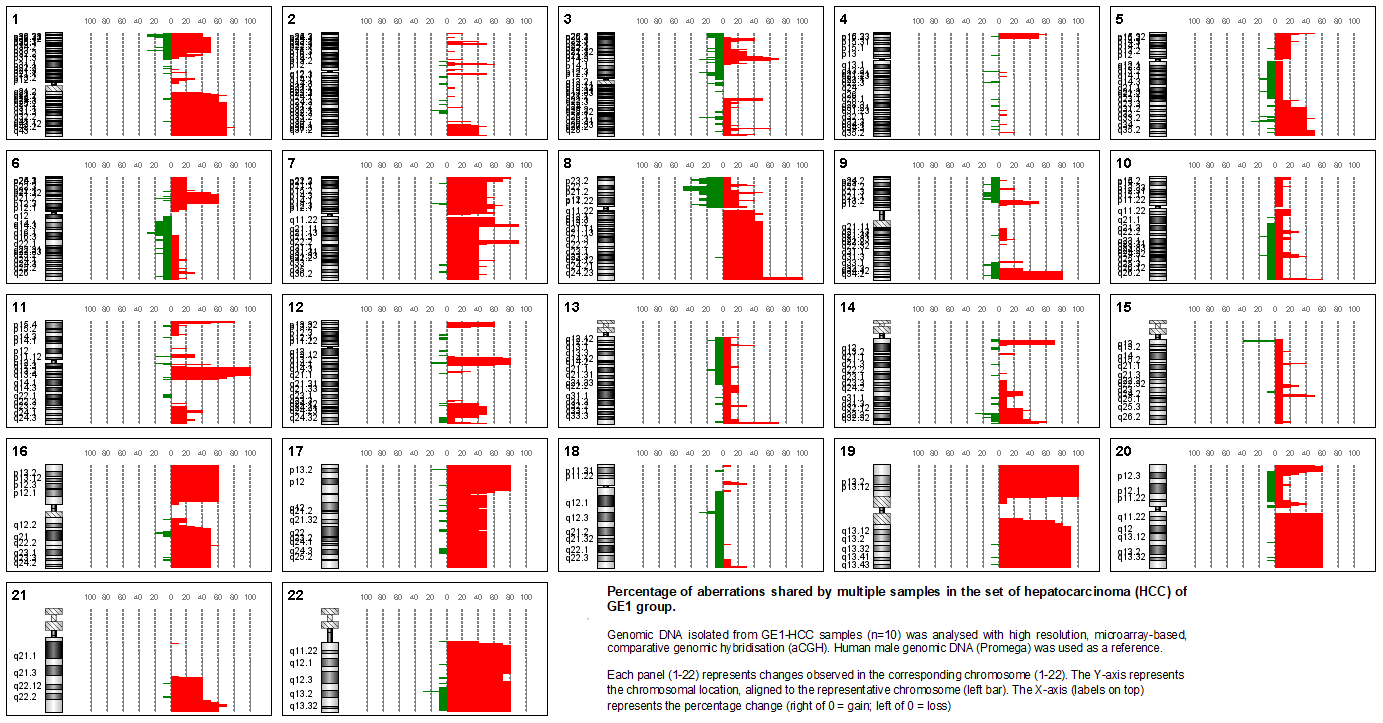

Supplement: Supplementary file 9 — Additional file 9. Percentage of aberrations shared by multiple samples in the set of hepatocarcinoma (HCC) of GE1 group. Genomic DNA isolated from GE1-HCC samples (n=10) was analysed using high resolution, microarray-based, comparative genomic hybridisation (aCGH). Human male genomic DNA (Promega) was used as a reference. [file 12920_2021_883_MOESM9_ESM.tif]

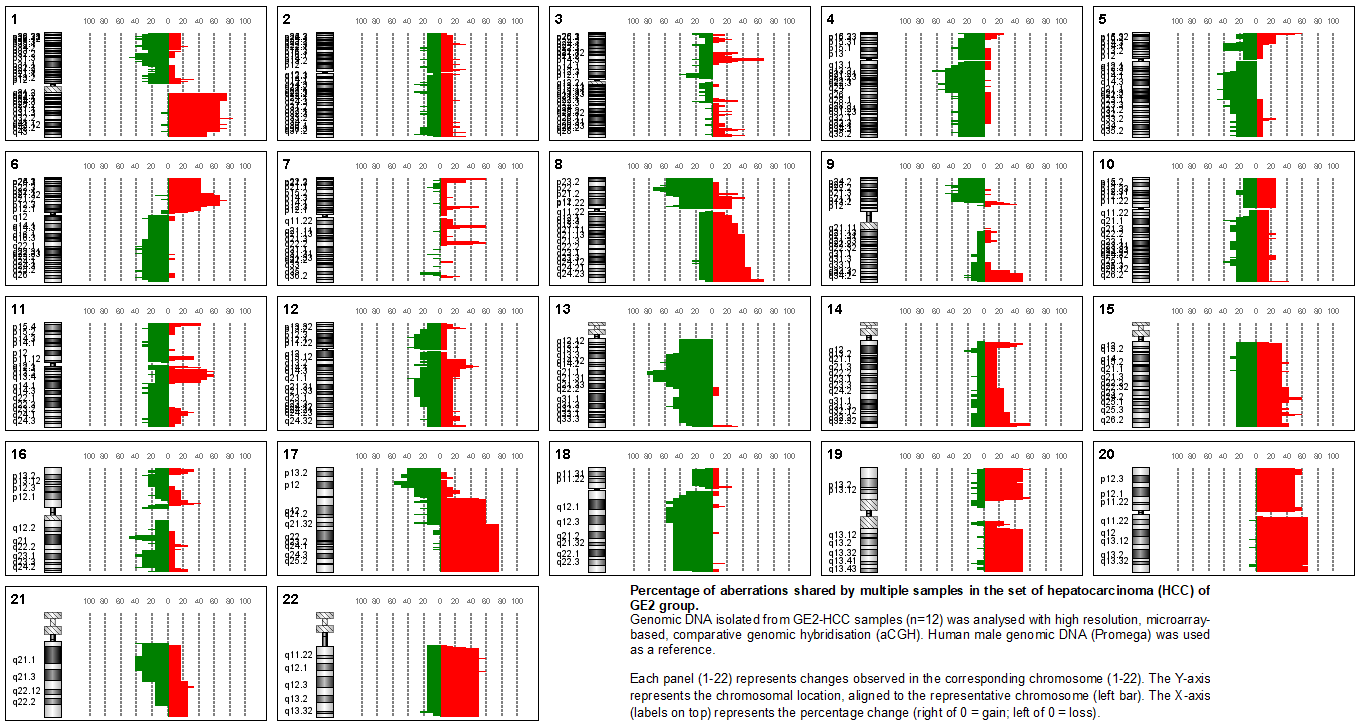

Supplement: Supplementary file 10 — Additional file 10. Percentage of aberrations shared by multiple samples in the set of hepatocarcinoma (HCC) of GE2 group. Genomic DNA isolated from GE2-HCC samples (n=12) was analysed using high resolution, microarray-based, comparative genomic hybridisation (aCGH). Human male genomic DNA (Promega) was used as a reference. [file 12920_2021_883_MOESM10_ESM.tif]
